# Synthesis and Biological Evaluation of Novel Biased Mu-Opioid Receptor Agonists

**DOI:** 10.3390/molecules29132961

**Published:** 2024-06-21

**Authors:** Yanhao Guo, Ruimin Yu, Tao Zhang, Fengxia Ren, Zixing Yu, Jingchao Cheng, Hongxin Jia, Weiguo Shi, Yatong Zhang

**Affiliations:** 1 College of Science, Hebei University of Science and Technology, Shijiazhuang 050018, China; guoyanhao123@126.com; 2 Center for Disease Control and Prevention of Central Theater Command, PLA, No.66 Heishitou Road, Beijing 100042, China; yrm0601@163.com; 3 Beijing Institute of Pharmacology & Toxicology, 27 Tai-Ping Road, Beijing 100850, Chinaren2019victory@126.com (F.R.);

**Keywords:** µOR-biased agonist, PZM21, safer opioids, β-arrestin-2 recruitment, structure activity relationship

## Abstract

This study explored the potential of a series of PZM21 analogues for pain treatment. Specifically, the hydroxyphenyl ring of PZM21 was replaced with a naphthyl ring, the thienyl ring was substituted with either a phenyl ring or furan rings, and the essential dimethylamine and urea groups were retained. These compounds aimed to enhance safety and minimize the adverse effects associated with opioid drugs. The research findings suggest that compound **6a** does not induce β-arrestin recruitment at low-nanomolar concentrations but exhibits significant analgesic effects in established mouse models. Compared to morphine, **6a** shows advantages in alleviating respiratory depression and minimizing physical dependence. Molecular docking studies underscore the pivotal role of the D147 amino acid residue in the analgesic mechanism of **6a**. Consequently, **6a** is a compelling candidate for the development of safer opioid analgesics and warrants further attention.

## 1. Introduction

Opioids are utilized to mitigate moderate-to-severe pain [1,2]; yet, their propensity for addiction, respiratory depression, and other severe side effects poses significant health risks and constrains their clinical use [3]. Therefore, the pursuit of developing opioids that preserve analgesic potency while diminishing the risks of respiratory depression and addiction is imperative.

Opioids exert their pharmacological effects by activating opioid receptors, which are a subclass within the G protein-coupled receptor family that is divided primarily into mu (μ), kappa, delta, and nociceptin subtypes [4]. Mu-opioid receptor (μOR) agonists are particularly efficacious in the management of moderate-to-severe pain, thereby fulfilling an indispensable role in the field of analgesic pharmacotherapy [5]. Studies have shown that μORs mediate analgesic effects through two distinct pathways: the G protein-mediated pathway, which plays a role in pain alleviation, and the β-arrestin pathway, which is implicated in the development of side effects [6,7,8].

**Figure 1:**
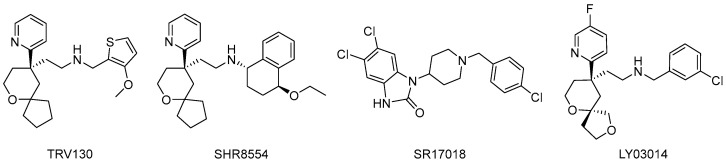
Structural formulae of related compounds.

Oliceridine (TRV130) is recognized as the world’s first G protein-biased μOR agonist, gaining approval from the U.S. Food and Drug Administration in 2020; it is distinguished by an innovative mechanism that notably mitigates the associated side effects of opioid medications [9,10,11,12,13,14,15,16]. Other compounds, namely SHR8554 [17], SR17018 [18], and LY03014 [19], are in various stages of clinical and preclinical development. These molecules have shown substantial analgesic efficacy with a rapid onset and are linked to a lower incidence of nausea and vomiting. The structural formulas of the pertinent compounds are depicted in Figure 1.

PZM21, which was identified from a dataset of approximately 3 million compounds through computer-aided drug design by Manglik [20], exhibits high selectivity for μOR, markedly activates the G protein-dependent signaling pathway, and exerts a diminished effect on the β-arrestin pathway [21,22]. In our previous study [23], we synthesized a series of PZM21 analogues by substituting the hydroxybenzene moiety of PZM21 with an aromatic naphthyl group, while keeping the pharmacophoric dimethyl amino and urea groups unchanged. We confirmed that the substitution of the phenolic hydroxyl group of PZM21 with a larger naphthyl group preserves µOR agonist activity, while it reduces selectivity for β-arrestin recruitment. Consequently, this moiety is postulated to be crucial for both analgesia and biased signaling.

**Figure 2:**
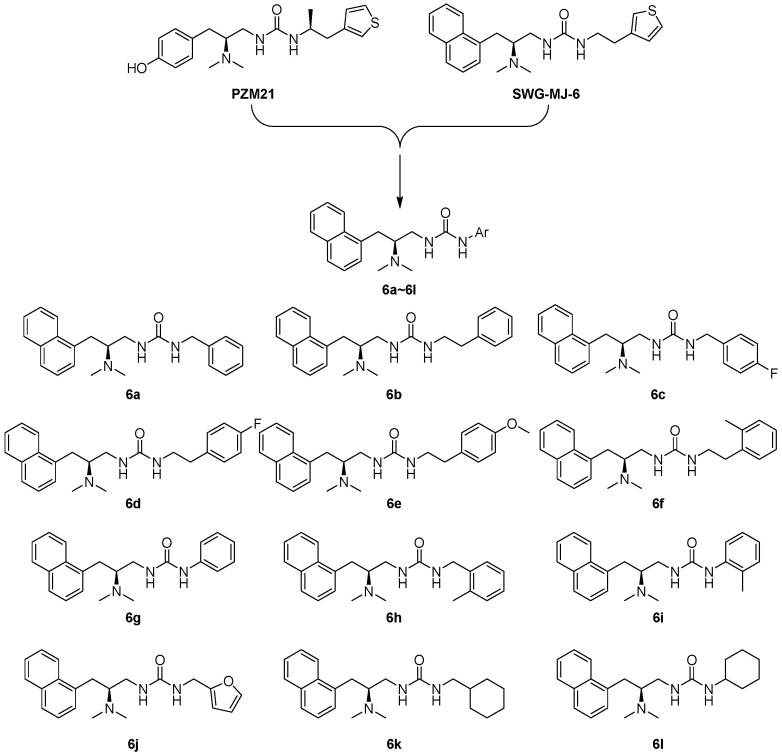
The design of novel PZM21 derivatives.

In this study, we investigate variations in the thiophene moiety. A series of PZM21 analogues was designed (Figure 2), and their biological activities were assessed both in vivo and in vitro, with the objective of determining whether these analogues could enhance the biosafety of opioids and minimize their side effects.

## 2. Results and Discussion

### 2.1. Synthesis

**Scheme 1:**
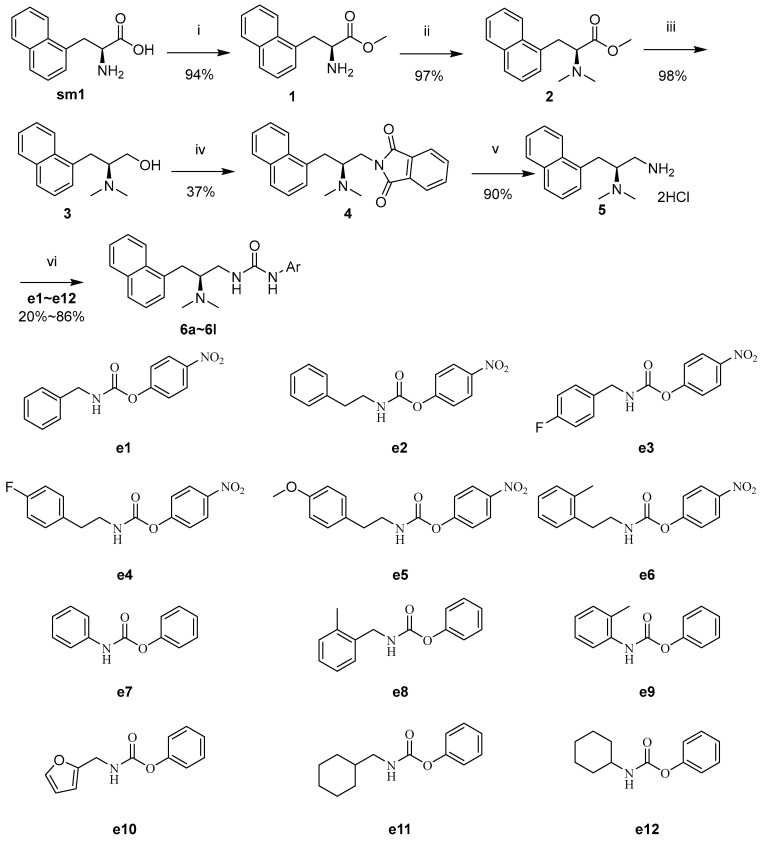
Synthesis of compounds **6a**–**l**. Reagents and conditions: (**i**) SOCl_2_, MeOH, ice bath then r.t., 12 h; (**ii**) STAB, 37% HCHO, ACN, r.t., 3 h; (**iii**) LiAlH_4_, THF, ice bath then r.t., 2 h; (**iv**) DIAD, PPh_3_, phthalimide, ice bath then r.t., 12 h; (**v**) NH_2_NH_2_, MeOH, reflux, 4 h; (**vi**) Et_3_N, ACN, 4 h.

The synthesis of compounds **6a**~**6l** was conducted in seven discrete steps, as illustrated in Scheme 1. The process initiated with the esterification of the unnatural amino acid **sm1**, resulting in intermediate **1**. Following this, intermediate **1** was reacted with formaldehyde (37% HCHO) and sodium triacetoxyborohydride (STAB) in an acetonitrile (ACN) solution, leading to the formation of intermediate **2**. Thereafter, intermediate **2** was reduced with lithium aluminum hydride (LiAlH_4_) in a tetrahydrofuran (THF) solution, yielding intermediate **3**. Following this step, intermediate **3** was reacted with triphenylphosphine (PPh_3_), phthalimide, and diisopropyl azodicarboxylate (DIAD) to effectuate the synthesis of intermediate **4** via the Mitsunobu reaction. The subsequent deprotection with hydrazine hydrate and methanol yielded the key intermediate **5**. Concurrently, raw materials **a1**~**a6** were reacted with 4-nitrophenyl chloroformate to yield **e1**~**e6**, respectively, while **a7**~**e12** underwent a similar conversion to provide **e7**~**e12** via reaction with phenyl chloroformate. The synthesis was finalized by the combination of intermediate **5** with **e1**~**e12**, yielding compounds **6a**~**6l**.

### 2.2. The Selective G_i/O_-Biased μOR Agonist Activities and the β-Arrestin Recruitment Assay In Vitro

#### 2.2.1. μOR G_i/o_-Mediated cAMP Inhibition

The µOR agonist activities of compounds **6a**~**6l** were assessed using a G_i/o_-protein signaling assay. To quantify the activation of µOR via the G_i/o_-mediated signaling cascade, we monitored the changes in intracellular cAMP levels, as the activity of adenosine cyclase is inhibited upon the receptor binding with its agonist, resulting in a reduced intracellular cAMP concentration. The G_i/o_-protein signaling assay revealed significant µOR agonist efficacy for the novel compounds **6a**~**6d**, **6f**, **6g**, **6h**, and **6j**, in addition to PZM21, as demonstrated in Table 1. The efficacies of compounds **6a** (half-maximal effective concentration (EC_50_ = 10.82 nM) and **6h** (EC_50_ = 13.12 nM) are similar to that of PZM21 (EC_50_ = 1.615 nM), while the other compounds exhibited lower potencies than that of PZM21.

#### 2.2.2. β-arrestin Recruitment Assay

The β-arrestin recruitment efficacies of compounds **6a**~**6l** were evaluated. As shown in Table 2, we observed a difference in the β-arrestin recruitment activities of morphine and PZM21. While morphine showed a significant level of recruitment with an EC_50_ value of 621.5 nM, it is important to interpret these results in the context of the maximal response elicited by each compound. PZM21, on the other hand, showed less efficacy in terms of the maximal level of β-arrestin recruitment it was able to achieve, despite having a lower EC_50_ value of 36.96 nM. This lower EC_50_ value indicates a higher potency for PZM21; however, the maximal response (efficacy) was lower than that of morphine. This distinction between potency and efficacy is critical for a comprehensive understanding of the pharmacological profiles of these compounds. Notably, compounds **6a**~**6l** were found to exhibit negligible recruitment activity at concentrations <100 μM.

### 2.3. In Vivo Antinociceptive Effects

#### 2.3.1. Evaluation of Antinociceptive Activities of the Novel Compounds in the Acetic Acid-Induced Writhing Assay

**Figure 3:**
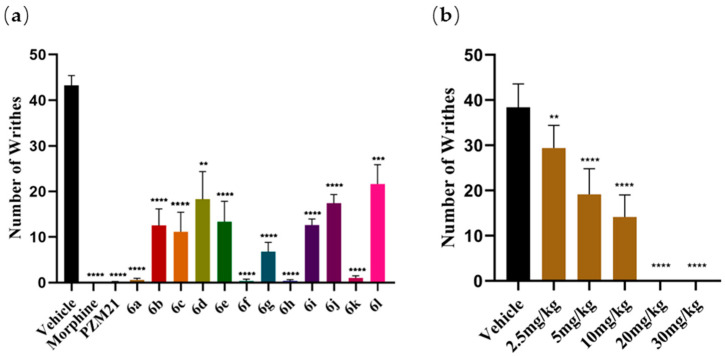
(**a**) Acetic acid-induced writhing test. (**b**) The antinociceptive activity of **6a** after i.v. injection at five different doses in the acetic acid-induced writhing test. The results are expressed as the mean ± standard error of the mean (*n* = 8). ** *p* < 0.01, *** *p* < 0.001, **** *p* < 0.0001 vs. vehicle (two-tailed Student’s *t* test).

In the acetic acid-induced writhing mouse model, morphine, PZM21, and the experimental compounds were administered via intravenous injection through the tail vein at doses of 2 mg/kg, 20 mg/kg, and 20 mg/kg, respectively. As shown in Figure 3a, compounds **6a**, **6f**, **6h**, and **6k** exhibited antinociceptive effects comparable to that of PZM21 when administered intravenously via the tail vein, with analgesia rates exceeding 97%. The antinociceptive effects in mice were assessed at incremental doses ranging from 2.5 mg/kg to 30 mg/kg, as indicated in Figure 3b. The results demonstrated a dose-dependent increase in efficacy, culminating in near-complete analgesia at a dose of 20 mg/kg.

#### 2.3.2. Antinociceptive Activities of the Novel Compounds in the Hot-Water Tail Withdrawal Test

**Figure 4:**
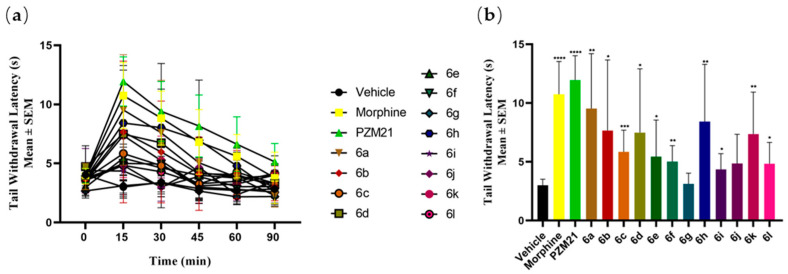
(**a**) The time course of antinociception of the test compounds in the hot-water tail-withdrawal test. (**b**) The antinociceptive effects of the test compounds in the hot-water tail-withdrawal assay were evaluated at the 15 min mark. The results are expressed as the mean ± standard error of the mean (*n* = 8). * *p* < 0.05, ** *p* < 0.01, *** *p* < 0.001, **** *p* < 0.0001 vs. vehicle (two-tailed Student’s *t* test).

In the hot-water tail-withdrawal test in mice, morphine, PZM21, and the experimental compounds were administered intravenously via the tail vein at doses of 2, 20, and 20 mg/kg, respectively. As shown in Figure 4a, all compounds reached their peak antinociceptive effects at 15 min post administration. Figure 4b shows the pain threshold data at the 15 min mark, with compounds **6a**, **6b**, and **6h** demonstrating significant antinociceptive action that was comparable to that of PZM21. The pain inhibition rates were 69.23% for PZM21, 50.19% for **6a**, 35.89% for **6b**, and 41.73% for **6h**.

#### 2.3.3. Antinociceptive Activities of the Novel Compounds in a Formalin-Induced Nociception Assay

**Figure 5:**
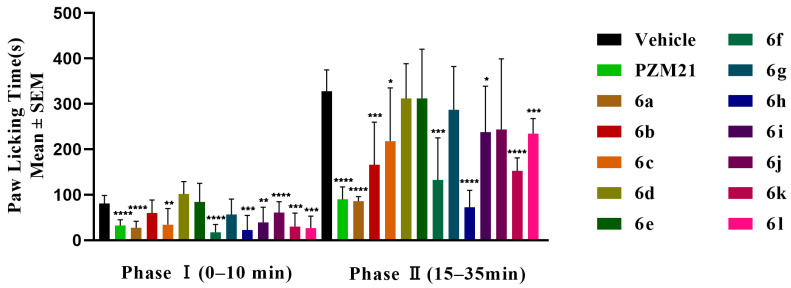
Formalin paw-licking test. The results are expressed as the mean ± standard error of the mean (*n* = 8). * *p* < 0.05, ** *p* < 0.01, *** *p* < 0.001, **** *p* < 0.0001 vs. vehicle; (two-tailed Student’s *t* test).

In the formalin-induced paw licking assay, PZM21, and the experimental compounds were administered intravenously via the tail vein at doses of 20 mg/kg, respectively. As shown in Figure 5, during the second phase of pain, compounds **6a**, **6f**, and **6h** exhibited prolonged antinociceptive effects. Comparison of these compounds with PZM21 revealed no significant differences. The antinociceptive efficacies were found to be 72.72% for PZM21, 73.80% for **6a**, 59.32% for **6f**, and 78.03% for **6h**.

### 2.4. In Vivo Adverse Effects Studies

**Figure 6:**
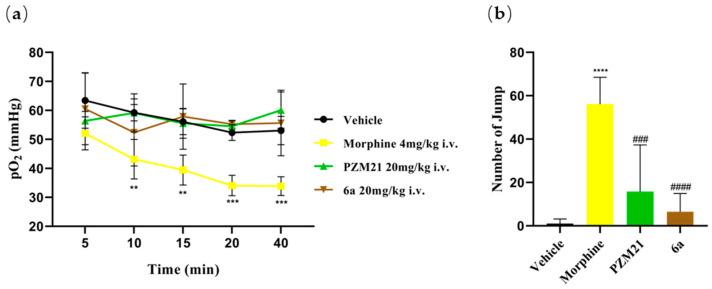
(**a**) The partial pressure of oxygen (pO_2_) of blood sampled from the eyes of mice at various time points was measured. (**b**) The number of jumps was counted over a 20 min period following naloxone injection. The results are expressed as the mean ± standard error of the mean (*n* = 8). ** *p* < 0.01, *** *p* < 0.001, **** *p* < 0.0001 vs. vehicle; ^###^ *p* < 0.01, ^####^ *p* < 0.0001 vs. morphine (two-tailed Student’s *t* test).

The respiratory depression of mice treated with **6a** was assessed using a blood gas analyzer. Compared to the vehicle group, neither the **6a** group nor the PZM21 group demonstrated any reduction in respiratory measures (Figure 6a), while the morphine-exposed mice exhibited a significant reduction in the partial pressure of oxygen level (measured in mmHg) in the blood. These data suggest that **6a** does not induce acute respiratory depression.

Moreover, the evaluation of physical dependence on **6a** in mice was performed using a precipitated withdrawal approach. Briefly, mice received naloxone (4 mg/kg, i.p.) following five consecutive days of test compound administration (i.e., morphine, PZM21, and the experimental compounds were administered intravenously via the tail vein at doses of 2 mg/kg, 20 mg/kg, and 20 mg/kg, respectively) to evaluate the physical dependence elicited by morphine, PZM21, and **6a**, respectively (Figure 6b). The mice administered morphine exhibited a significantly greater frequency of jumping compared to those receiving the vehicle, PZM21, or **6a**, suggesting that morphine induces significant physical dependence and withdrawal symptoms compared to the vehicle. In contrast, neither **6a** nor PZM21 elicited a significant increase in withdrawal behaviors compared with vehicle treatment, indicating that **6a** does not induce physical dependence or withdrawal symptoms in mice.

### 2.5. Molecular Modeling

**Figure 7:**
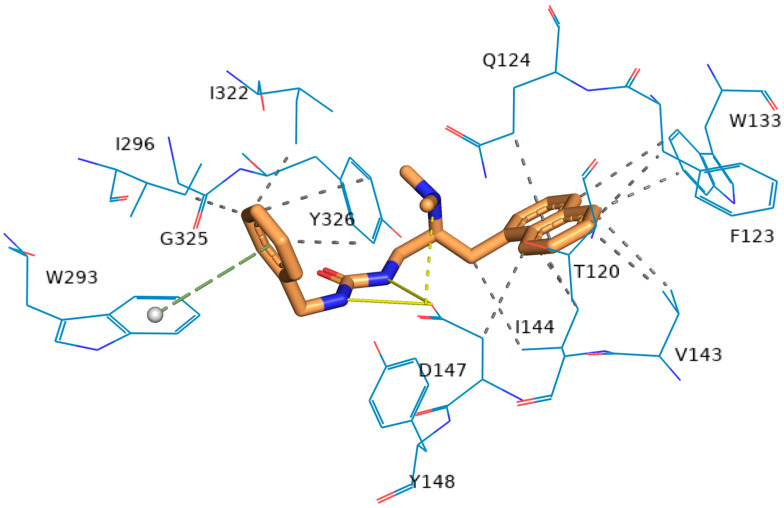
Structure of the μOR-G_i/o_ complex bound to **6a**. The molecule **6a** is presented as a thin stick model. The residues are shown as line models.

To explore the binding affinity of compound **6a** for the μOR-G_i/O_ pathway protein, AutoDock Vina was employed to conduct a preliminary docking study on **6a** with the μOR-G_i/O_ protein (PDB ID: 7SBF), as depicted in Figure 7. Compound 6a engaged in hydrophobic interactions with the residues D147, F123, I144, I296, I322, Q124, V143, and Y326, engaged in π-stacking with residue W293, and formed salt bridges with residue D147. Residue D147 formed strong hydrogen bonds with compound **6a**, as evidenced by the 2.3 Å distance between them, and the binding affinity was found to be −9.8 kcal/mol, indicating compound **6a**’s significant potential for interaction with the μOR-G_i/O_ protein. Thus, compound **6a** warrants further investigation within the μOR-G_i/O_ protein pathway.

## 3. Materials and Methods

### 3.1. General Information

All reactions were routinely monitored using thin layer chromatography (TLC) on silica gel plates (GF254) and visualized with a UV lamp (λ = 254 nm). The pipettes used in the experiments were supplied by DLAB Scientific Co., Ltd. (Beijing, China). ^1^H NMR and ^13^C NMR spectra were recorded on a spectrometer operating at 600 MHz and 151 MHz, respectively, using DMSO–*d*_6_ or CDCl_3_ as the solvent and TMS as an internal standard. High resolution mass spectra (HR MS) were obtained using an Agilent (Santa Clara, CA, USA) 6210 ESI/TOF mass spectrometer. 

### 3.2. Chemical and Reagents

In the experiments conducted, the reagents and chemicals were obtained from Energy Chemical. The reagents and solvents used were of commercial grade and employed without further purification.

### 3.3. Animals

In the current experiment, SPF-grade male or female CD-1 mice weighing 23–26 g from the Beijing SPF (Beijing, China) Biotechnology Co., Ltd., with the license number (SCXK Jing 2019-0010), were used. The mice were group-housed in a controlled temperature environment and subjected to a 12 h light/dark cycle (lights on from 07:00 to 19:00). Food and water were provided ad libitum. All animal procedures were conducted in accordance with the policies and recommendations of the International Association for the Study of Pain and the National Health and Animal Welfare Committee of the Beijing Institute of Pharmacology, with the aim of minimizing the number of animals used and their suffering.

### 3.4. Chemistry

#### 3.4.1. Synthesis of **e1**~**e6**

Various substituted amines, **a1** or **a2** or **a3** or **a4** or **a5** or **a6** (5 g, 1.0 eq), Et_3_N (3.4 eq), and THF (100 mL) were mixed in a reaction flask and cooled to 0 °C with an ice-salt bath. 4-Nitrophenyl chloroformate (1.15 eq) was added to the mixture and stirred for 8 h at room temperature. When the reaction was complete, 25 mL of dichloromethane was added to the reaction mixture and the mixture was filtered. The organic phase was washed twice sequentially with 10 mL of saturated NaHCO_3_, NH_4_Cl, and NaCl solutions. The organic layer was dried over Na_2_SO_4_, filtered, and concentrated by rotary evaporation. The product was purified by column chromatography (4:1 petroleum ether: dichloromethane) to afford compounds **e1**~**e6** (2.6 g~5.1 g) as white solid with a yield of 21%~44%. 

#### 3.4.2. Synthesis of **e7**~**e12**

Various substituted amines, **a7** or **a8** or **a9** or **a10** or **a11** or **a12** (1 g, 1.0 eq), Et_3_N (3.4 eq), and THF (20 mL) were mixed in a three-necked reaction flask and the solution was stirred in an ice-salt bath to 0 °C. Phenyl chloroformate (1.05 eq) was added to the mixture and stirred for 8 h at room temperature. Then, 5 mL of dichloromethane was added to the reaction mixture and stirred for 10 min, followed by filtering of the mixture. The organic phase was washed twice sequentially with 10 mL of saturated NaHCO_3_, NH_4_Cl, and NaCl solutions. The organic layer was dried over Na_2_SO_4_, filtered and concentrated. The product was recrystallized using a mixture of dichloromethane and n-hexane to obtain a white solid **e7**~**e12** (0.69 g~1.77 g) with a yield of 33~85%.

#### 3.4.3. Synthesis of **5**

Twenty-five grams of 3-(1-naphthyl)-L-alanine (0.116 mol, 1.0 eq) and 500 mL of MeOH were mixed and stirred with a magnetic stirrer. We slowly added 69.1 g of SOCl_2_ (0.581 mol, 5.0 eq), keeping the temperature below 5 °C. The reaction was carried out at room temperature for 12 h. The reaction mixture was concentrated under reduced pressure until no solvent remained, yielding a light yellow solid. It was desalted with a 10% sodium hydroxide aqueous solution to finally obtain a brown oily substance (intermediate **1**) 26.2 g (yielding 94%).

Then, 26 g of intermediate **1** (0.114 mol, 1.0 eq), 130.0 g of 37% formaldehyde solution (0.160 mol, 14.0 eq), and 520 mL of acetonitrile were mixed and stirred at room temperature. Next, 96.95 g of sodium triacetoxyborohydride (0.456 mol, 4.0 eq) was added to the reaction mixture and stirred for 3 h. The acetonitrile was removed under reduced pressure; the mixture was extracted with ethyl acetate (500 mL × 3); the organic phases were combined, washed with saturated brine (260 mL); the solvent was removed to obtain a brown oily substance (intermediate 2) weighing 28.7 g(yielding 97%).

Intermediate **2** (18.7 g, 0.073 mol, 1.0 eq) and THF 600 mL were mixed and cooled to 0 °C with an ice-salt bath. LiAlH_4_ 8.3 g (0.219 mol, 3.0 eq) was added to the mixture and kept stirred for 2 h. Then, 8.3 g of water and 100 mL of a 10% NaOH aqueous solution were added to the mixture, stirred for half an hour, and filtered. The filter cake was washed with 40 mL of THF. The filtrate was concentrated to remove THF, ethyl acetate (200 mL) was added, and it was washed with saturated brine (100 mL). The solvent was removed under reduced pressure to obtain a white solid (intermediate **3**) weighing 16.34 g (yielding 98%).

Intermediate **3** (16.34 g, 0.071 mol, 1.0 eq), triphenylphosphine (22.43 g, 0.085 mol, 1.2 eq), phthalimide (16.2 g, 0.085 mol, 1.2 eq), and THF 400 mL were mixed and stirred until the solution became clear. DIAD (20.17 g, 0.099 mol, 1.4 eq) was slowly added to the mixture and stirred overnight. Then, 160 mL of 2 M hydrochloric acid was added and the mixture was extracted with ethyl acetate (100 mL × 2). The product was purified by column chromatography (40:1 DCM: MeOH) to afford intermediate **4** as a brown oil weighing 9.56 g, with a yield of 37%. 

Intermediate **4** (9.56 g, 0.027 mol, 1.0 eq), 85% hydrazine hydrate (5.5 g, 0.093 mol, 3.5 eq), and methanol (100 mL) were mixed and heated to reflux for 4 h. The reaction mixture was concentrated under reduced pressure until no solvent remained. Then, ethyl acetate (50 mL) was added and the mixture was stirred for 30 min. The filtrate was concentrated. Intermediate **5** 7.2 g was obtained via crystallize using a solution of 2 M hydrochloric acid in ethyl acetate (20 mL) as a pale yellow solid with a yield of 90%. MS: 229.17 (M + H)^+^.

#### 3.4.4. Synthesis of **6a**~**6l**

Intermediate **5** (0.40 g, 1.32 mmol, 1.0 eq), triethylamine (0.45 g, 4.51 mmol, 3.4 eq), and acetonitrile (6 mL) were mixed and stirred. The mixture was heated to 60 °C for 30 min, followed by the dropwise addition of a solution of compound (**e1** or **e2** or **e3** or **e4** or **e5** or **e6** or **e7** or **e8** or **e9** or **e10** or **e11** or **e12**, 1.46 mmol, 1.1 eq) in acetonitrile (2 mL). The mixture was stirred at 85 °C for 4 h. The reaction mixture was concentrated under reduced pressure to remove the solvent. The final product was purified by column chromatography (50:1 DCM:MeOH) to afford a yellow-brown oily substance **6a**~**6l** (0.10 g~0.42 g) with a yield of (20.49~87.50%). The HPLC purity of **6a**~**6l** was >95%.

*(S)-1-benzyl-3-(2-(dimethylamino)-3-(naphthalen-1-yl)propyl) urea* (**6a**): ^1^H NMR (600 MHz, DMSO-*d*_6_) *δ* 8.07 (d, *J* = 8.3 Hz, 1H), 7.93 (d, *J* = 8.1 Hz, 1H), 7.78 (d, *J* = 8.1 Hz, 1H), 7.56–7.50 (m, 2H), 7.43 (t, *J* = 7.4 Hz, 1H), 7.38 (br d, *J* = 6.9 Hz, 1H), 7.27–7.25 (m, 2H), 7.19–7.17 (m, 3H), 6.54 (t, *J* = 6.0 Hz, 1H), 5.81–5.80 (m, 1H), 4.11 (d, *J* = 6.0 Hz, 2H), 3.37–3.34 (m, 1H), 3.07–2.97 (m, 2H), 2.87–2.81 (m, 2H), 2.39 (s, 6H). ^13^C NMR (150 MHz, DMSO-*d*_6_) *δ* 157.8, 140.9, 136.0, 133.5, 131.6, 128.7, 128.1, 127.5, 126.9, 126.6, 126.5, 126.0, 125.5, 123.5, 64.0, 42.8, 40.0, 39.5, 28.5. HR-ESI-MS *m*/*z* 362.2227 [M + H]^+^ (calcd. for C_23_H_28_N_3_O, 362.2232).
*(S)-1-(2-(dimethylamino)-3-(naphthalen-1-yl)propyl)-3-phenethyl urea* (**6b**): ^1^H NMR (600 MHz, DMSO-*d*_6_) *δ* 8.07 (d, *J* = 8.4 Hz, 1H), 7.93 (d, *J* = 8.1 Hz, 1H), 7.79 (d, *J* = 8.1 Hz, 1H), 7.58–7.49 (m, 2H), 7.46–7.41 (m, 1H), 7.38 (br d, *J* = 6.8 Hz, 1H), 7.26–7.23 (m, 2H), 7.18–7.12 (m, 3H), 6.08 (t, *J* = 5.4 Hz, 1H), 5.74 (br s, 1H), 3.37–3.34 (m, 1H), 3.13 (dd, *J* = 13.7, 6.6 Hz, 2H), 3.05–2.93 (m, 2H), 2.88–2.79 (m, 2H), 2.60 (t, *J* = 7.3 Hz, 2H), 2.38 (s, 6H). ^13^C NMR (150 MHz, DMSO-*d*_6_) *δ* 157.7, 139.7, 136.0, 133.6, 131.6, 128.7, 128.6, 128.2, 127.6, 126.6, 126.0, 125.9, 125.5, 125.5, 123.5, 64.1, 40.8, 40.0, 39.3, 36.2, 28.5. HR-ESI-MS *m*/*z* 376.2383 [M + H]^+^ (calcd. for C_24_H_30_N_3_O, 376.2389).
*(S)-1-(2-(dimethylamino)-3-(naphthalen-1-yl)propyl)-3-(4-fluoro benzyl) urea* (**6c**): ^1^H NMR (600 MHz, DMSO-*d*_6_) *δ* 8.07 (d, *J* = 8.3 Hz, 1H), 7.93 (d, *J* = 7.9 Hz, 1H), 7.79 (d, *J* = 8.1 Hz, 1H), 7.57–7.49 (m, 2H), 7.45–7.36 (m, 2H), 7.22–7.17 (m, 2H), 7.10–7.05 (m, 2H), 6.58–6.53 (m, 1H), 5.79 (br s, 1H), 4.08 (d, *J* = 5.9 Hz, 2H), 3.37–3.29 (m, 1H), 3.06–2.92 (m, 2H), 2.90–2.78 (m, 2H), 2.39 (s, 6H). ^13^C NMR (150 MHz, DMSO-*d*_6_) *δ* 161.0 (d, *J* = 241.8 Hz), 157.8, 137.1, 133.5, 131.6, 128.8 (d, *J* = 8.0 Hz), 128.7, 127.6, 126.6, 126.6, 126.0, 125.5 (d, *J* = 2.7 Hz), 123.5, 114.8 (d, *J* = 21.1 Hz), 64.0, 42.1, 40.1, 40.0, 28.4. HR-ESI-MS *m*/*z* 380.2133 [M + H]^+^ (calcd. for C_23_H_27_FN_3_O, 380.2138).
*(S)-1-(2-(dimethylamino)-3-(naphthalen-1-yl)propyl)-3-(4-fluoro phenethyl) urea* (**6d**): ^1^H NMR (600 MHz, DMSO-*d*_6_) *δ* 8.07 (d, *J* = 8.3 Hz, 1H), 7.93 (d, *J* = 7.7 Hz, 1H), 7.79 (d, *J* = 8.1 Hz, 1H), 7.58–7.49 (m, 2H), 7.46–7.41 (m, 1H), 7.38 (br d, *J* = 6.8 Hz, 1H), 7.20–7.15 (m, 2H), 7.09–7.03 (m, 2H), 6.09–6.04 (m, 1H), 5.71 (br s, 1H), 3.38–3.29 (m, 1H), 3.11 (dd, *J* = 13.3, 6.7 Hz, 2H), 3.06–2.98 (m, 1H), 2.98–2.78 (m, 1H), 2.88–2.78 (m, 2H), 2.58 (t, *J* = 7.2 Hz, 2H), 2.38 (s, 6H). ^13^C NMR (150 MHz, DMSO-*d*_6_) *δ* 160.7 (d, *J* = 241.4 Hz), 157.7, 135.9, 135.8, 133.5, 131.6, 130.3 (d, *J* = 7.6 Hz), 128.7, 127.6, 126.6, 126.0, 125.5 (d, *J* = 3.0 Hz), 123.5, 114.9 (d, *J* = 21.0 Hz), 64.1, 40.8, 40.1, 35.2, 28.4. HR-ESI-MS *m*/*z* 394.2289 [M + H]^+^ (calcd. for C_24_H_29_FN_3_O, 394.2295).
*(S)-1-(2-(dimethylamino)-3-(naphthalen-1-yl)propyl)-3-(4-methoxy phenethyl) urea* (**6e**): ^1^H NMR (600 MHz, DMSO-*d*_6_) *δ* 8.07 (d, *J* = 8.3 Hz, 1H), 7.93 (d, *J* = 7.8 Hz, 1H), 7.79 (d, *J* = 8.1 Hz, 1H), 7.58–7.49 (m, 2H), 7.47–7.41 (m, 1H), 7.38 (br d, *J* = 6.7 Hz, 1H), 7.07–7.04 (m, 2H), 6.84–6.78 (m, 2H), 6.04 (t, *J* = 4.8 Hz, 1H), 5.72 (br s, 1H), 3.69 (s, 3H), 3.39–3.27 (m, 1H), 3.08 (dd, *J* = 13.5, 6.7 Hz, 2H), 3.05–2.99 (m, 1H), 2.99–2.89 (m, 1H), 2.90–2.77 (m, 2H), 2.52 (t, *J* = 7.3 Hz, 2H), 2.38 (s, 6H). ^13^C NMR (150 MHz, DMSO-*d*_6_) *δ* 157.5, 133.5, 131.6, 129.5, 128.7, 127.6, 126.6, 126.0, 125.5, 125.5, 123.5, 113.7, 64.1, 54.9, 41.1, 40.1, 35.2, 28.5. HR-ESI-MS *m*/*z* 406.2489 [M + H]^+^ (calcd. for C_25_H_32_N_3_O_2_, 406.2495).
*(S)-1-(2-(dimethylamino)-3-(naphthalen-1-yl) propyl)-3-(2-methyl phenethyl) urea* (**6f**): ^1^H NMR (600 MHz, DMSO-*d*_6_) *δ* 8.08 (d, *J* = 8.4 Hz, 1H), 7.93 (d, *J* = 7.8 Hz, 1H), 7.79 (d, *J* = 8.1 Hz, 1H), 7.58–7.49 (m, 2H), 7.47–7.42 (m, 1H), 7.39 (br d, *J* = 6.9 Hz, 1H), 7.14–7.08 (m, 1H), 7.07–7.03 (m, 3H), 6.15 (t, *J* = 5.4 Hz, 1H), 5.73 (br s, 1H), 3.39–3.33 (m, 1H), 3.07 (dd, *J* = 14.6, 6.2 Hz, 2H), 3.05–3.00 (m, 1H), 2.99–2.93 (m, 1H), 2.90–2.78 (m, 2H), 2.62–2.56 (m, 2H), 2.40 (s, 6H), 2.22 (s, 3H). ^13^C NMR (150 MHz, DMSO-*d*_6_) *δ* 157.8, 137.8, 135.9, 133.6, 131.6, 129.9, 129.0, 128.7, 127.6, 126.6, 126.0, 126.0, 125. 8, 125.5, 123.5, 64.1, 40.1, 39.3, 33.8, 28.5, 18.9. HR-ESI-MS *m*/*z* 390.2540 [M + H]^+^ (calcd. for C_25_H_32_N_3_O, 390.2545).
*(S)-1-(2-(dimethylamino)-3-(naphthalen-1-yl) propyl)-3-phenyl urea* (**6g**): ^1^H NMR (600 MHz, DMSO-*d*_6_) *δ* 8.67 (s, 1H), 8.09 (d, *J* = 8.4 Hz, 1H), 7.93 (d, *J* = 8.1 Hz, 1H), 7.80 (d, *J* = 8.1 Hz, 1H), 7.58–7.54 (m, 1H), 7.53–7.49 (m, 1H), 7.45 (t, *J* =7.5 Hz, 1H), 7.41 (br d, *J* = 6.9 Hz, 1H), 7.29 (d, *J* = 8.0 Hz, 2H), 7.15 (t, *J* = 8.0 Hz, 2H), 6.83 (t, *J* = 7.3 Hz, 1H), 6.05 (br d, *J* = 5.8 Hz, 1H), 3.42 (dd, *J* = 13.3, 3.2 Hz, 1H), 3.10 (ddd, *J* = 12.0, 7.1, 4.5 Hz, 1H), 3.04–2.96 (m, 1H), 2.95–2.88 (m, 1H), 2.87–2.80 (m, 1H), 2.43 (s, 6H). ^13^C NMR (150 MHz, DMSO-*d*_6_) *δ* 154.9, 140.6, 135.9, 133.6, 131.6, 128.8, 128. 6, 127.6, 126.7, 126.0, 125.6, 125.5, 123.5, 120.8, 117.4, 63.9, 40.1, 39.9, 28.0. HR-ESI-MS *m*/*z* 348.2070 [M + H]^+^ (calcd. for C_22_H_26_N_3_O, 348.2076).
*(S)-1-(2-(dimethylamino)-3-(naphthalen-1-yl) propyl)-3-(2-methyl benzyl) urea* (**6h**): ^1^H NMR (600 MHz, DMSO-*d*_6_) *δ* 8.08 (d, *J* = 8.3 Hz, 1H), 7.93 (d, *J* = 7.6 Hz, 1H), 7.79 (d, *J* = 8.1 Hz, 1H), 7.59–7.48 (m, 2H), 7.47–7.41 (m, 1H), 7.39 (br d, *J* = 6.8 Hz, 1H), 7.17–7.06 (m, 4H), 6.44 (t, *J* = 5.5 Hz, 1H), 5.83 (br s, 1H), 4.14–4.05 (m, 2H), 3.42–3.33 (m, 1H), 3.09–2.95 (m, 2H), 2.94–2.79 (m, 2H), 2.40 (s, 6H), 2.21 (s, 3H). ^13^C NMR (150 MHz, DMSO-*d*_6_) *δ* 157.7, 138.4, 136.0, 135.3, 133.5, 131.6, 129.8, 128.7, 127.5, 127.2, 126.6, 126.5, 126.0, 125.6, 125.5, 125.5, 123.5, 64.1, 40.9, 40.0, 28.5, 18.5. HR-ESI-MS *m*/*z* 376.2383 [M + H]^+^ (calcd. for C_24_H_30_N_3_O, 376.2389).
*(S)-1-(2-(dimethylamino)-3-(naphthalen-1-yl) propyl)-3-(o-tolyl) urea* (**6i**): ^1^H NMR (600 MHz, DMSO-*d*_6_) *δ* 8.08 (d, *J* = 8.4 Hz, 1H), 7.93 (d, *J* = 8.0 Hz, 1H), 7.83 (s, 1H), 7.80 (d, *J* = 8.0 Hz, 1H), 7.70 (d, *J* = 8.1 Hz, 1H), 7.58–7.54 (m, 1H), 7.53–7.49 (m, 1H), 7.47–7.43 (m, 1H), 7.41 (br d, *J* = 6.8 Hz, 1H), 7.06 (d, *J* = 7.4 Hz, 1H), 7.03–6.99 (m, 1H), 6.82 (t, *J* = 7.4 Hz, 1H), 6.53 (br d, *J* = 4.8 Hz, 1H), 3.42 (dd, *J* = 13.4, 3.5 Hz, 1H), 3.18–3.10 (m, 1H), 3.09–3.01 (m, 1H), 2.98–2.89 (m, 1H), 2.88–2.82 (m, 1H), 2.44 (s, 6H), 2.14 (s, 3H). ^13^C NMR (150 MHz, DMSO-*d*_6_) *δ* 155.2, 138.3, 135.9, 133.6, 131.6, 130.0, 128.8, 127.6, 126.7, 126.6, 126.0, 125.9, 125.5, 125.5, 123.5, 121.7, 120.6, 64.0, 40.0, 39.3, 28.3, 18.0. HR-ESI-MS *m*/*z* 362.2227 [M + H]^+^ (calcd. for C_23_H_28_N_3_O, 362.2232).
*(S)-1-(2-(dimethylamino)-3-(naphthalen-1-yl) propyl)-3-(furan-2-yl methyl) urea* (**6j**): ^1^H NMR (600 MHz, DMSO-*d*_6_) *δ* 8.07 (d, *J* = 8.3 Hz, 1H), 7.93 (d, *J* = 7.7 Hz, 1H), 7.78 (d, *J* = 8.1 Hz, 1H), 7.58–7.48 (m, 3H), 7.46–7.41 (m, 1H), 7.38 (br d, *J* = 6.8 Hz, 1H), 6.47 (t, *J* = 5.6 Hz, 1H), 6.33 (dd, *J* = 3.3, 1.8 Hz, 1H), 6.12 (m, 1H), 5.82 (br d, *J* = 4.3 Hz, 1H), 4.09 (d, *J* = 5.7 Hz, 2H), 3.35 (dd, *J* = 12.8, 3.1 Hz, 1H), 3.04 (ddd, *J* = 11.9, 7.1, 4.6 Hz, 1H), 3.01–2.93 (m, 1H), 2.91–2.79 (m, 2H), 2.38 (s, 6H). ^13^C NMR (150 MHz, DMSO-*d*_6_) *δ* 157.4, 153.7, 141.8, 136.0, 133.5, 131.6, 128.7, 127.5, 126.6, 126.0, 125.5, 125.5, 123.5, 110.3, 106.0, 64.0, 40.1, 40.0, 36.2, 28.4. HR-ESI-MS *m*/*z* 352.2020 [M + H]^+^ (calcd. for C_21_H_26_N_3_O_2_, 352.2025).
*(S)-1-(cyclohexylmethyl)-3-(2-(dimethylamino)-3-(naphthalen-1-yl) propyl) urea* (**6k**): ^1^H NMR (600 MHz, CDCl_3_) *δ* 7.98 (d, *J* = 8.4 Hz, 1H), 7.83–7.78 (m, 1H), 7.68 (d, *J* = 8.2 Hz, 1H), 7.50–7.42 (m, 2H), 7.31–7.35 (m, 1H), 7.28–7.26 (m, 1H), 5.44 (br s, 1H), 5.19 (br s, 1H), 3.46 (dd, *J* = 13.6, 2.6 Hz, 1H), 3.20–3.09 (m, 1H), 3.07–2.99 (m, 2H), 2.85 (t, *J* = 6.3 Hz, 2H), 2.78–2.73 (m, 1H), 2.42 (s, 6H), 2.11–1.98 (m, 2H), 1.67–1.58 (m, 3H), 1.32–1.25 (m, 1H), 1.18–1.05 (m, 3H), 0.85–0.75 (m, 2H). ^13^C NMR (150 MHz, CDCl_3_) *δ* 158.9, 134.7, 134.1, 131.8, 129.0, 127.8, 127.3, 126.0, 125.6, 125.6, 123.3, 64.3, 46.7, 40.5, 40.1, 38.4, 32.2, 30.8, 26.5, 25.9. HR-ESI-MS *m*/*z* 368.2696 [M + H]^+^ (calcd. for C_23_H_34_N_3_O, 368.2702).
*(S)-1-cyclohexyl-3-(2-(dimethylamino)-3-(naphthalen-1-yl) propyl) urea* (**6l**): ^1^H NMR (600 MHz, CDCl_3_) *δ* 7.99 (d, *J* = 8.4 Hz, 1H), 7.84 (d, *J* = 8.0 Hz, 1H), 7.71 (d, *J* = 8.1 Hz, 1H), 7.52–7.44 (m, 2H), 7.38–7.34 (m, 1H), 7.30 (br d, *J* = 6.9 Hz, 1H), 5.21 (br s, 1H), 4.77 (br s, 1H), 3.51 (dd, *J* = 13.6, 3.1 Hz, 1H), 3.34–3.41 (m, 1H), 3.20–3.13 (m, 1H), 3.11–3.01 (m, 2H), 2.79 (dd, *J* = 13.6, 9.9 Hz, 1H), 2.47 (s, 6H), 1.85–1.77 (m, 2H), 1.66–1.59 (m, 2H), 1.57–1.51 (m, 1H), 1.32–1.22 (m, 2H), 1.13–1.05 (m, 1H), 1.04–0.96 (m, 2H). ^13^C NMR (150 MHz, CDCl_3_) *δ* 158.0, 134.7, 134.2, 131.9, 129.2, 127.9, 127.5, 126.2, 125.7, 125.7, 123.3, 64.4, 49.0, 40.7, 40.2, 33.9, 29.1, 25.7, 25.0. HR-ESI-MS *m*/*z* 354.2542 [M + H]^+^ (calcd. for C_22_H_32_N_3_O, 354.2545).

### 3.5. In Vitro Pharmacology

#### 3.5.1. μOR G_i/o_-Mediated Cyclic Adenosine Monophosphate (cAMP) Inhibition Assay

The inhibition assay was performed as described previously [23]. In brief, tested compound dilutions (with final concentrations of 100 µM, 50 µM, 10 µM, 1 µM, 500 nM, 100 nM, 50 nM, 10 nM, 1 nM, and 100 pM) were produced in saline or DMSO in triplicate. CHO/μOR cells were suspended in the assay buffer and counted, maintaining a cell density of 2000 cells per well, with 5 µL from each well seeded into a 384-well plate. Subsequently, 5 µL of the tested compound solution per well was dispensed into the plate, which was then placed in a 25 °C incubator and incubated in the dark for 30 min. Next, 10 µL of cAMP standard working solution was added to the empty wells of the plate, followed by the addition of 5 µL of Eu-cAMP and Anti-cAMP per well. The plate was subsequently placed back in a 25 °C incubator and further incubated in the dark for 1 h. LANCE readings were conducted using the Envision system (excitation: 320 nm, emission: 615 nm and 665 nm).

##### Cell Culture

CHO cells expressing the human μ-opioid receptor were cultivated for the assessment of compound effects. Cultured in F-12 medium supplemented at 37 °C and 5% CO_2_, these cells exhibit consistent μOR expression. Cells collected during the exponential phase of growth were dissociated without enzymes to preserve receptor integrity, resuspended in assay buffer, and counted to achieve a uniform concentration. Cell density was adjusted to 2000 cells per well in a 384-well plate, promoting uniform distribution and optimal receptor presence. Post-seeding at 25 °C in darkness facilitated cell adherence and stabilization prior to compound introduction. Compounds were added in serial dilutions, with meticulous dispensing techniques ensuring dose uniformity. Following a 1 h incubation at 25 °C, TR-FRET reagents were applied to measure cAMP levels, indicative of μOR activity. The Envision system recorded these levels, quantifying compound inhibitory effects. Meticulous aseptic techniques and vigilant monitoring were imperative throughout to guarantee data precision from the assay.

#### 3.5.2. β-Arrestin Recruitment Assay

In this assay, optical signal values were detected employing the PathHunter^®^ (DiscoverX, Shanghai, China) Detection Kit assay method. The agonistic activity of tested compounds on the human μOR β-arrestin2 pathway was quantified using a β-galactosidase-based enzyme fragment complementation assay. In brief, tested compound dilutions (with final concentrations of 100 μM, 50 μM, 10 μM, 1 μM, 500 nM, 100 nM, 50 nM, 10 nM, 1 nM, and 100 pM) were produced in saline or DMSO in triplicate. Next, 100 nL of the tested compound solution per well was dispensed into a 384-well cell culture plate. The U2OS/human μOR β-arrestin cells were resuspended in Opti-MEM and counted to achieve a cell density of 4000 cells per well, and 20 μL from each well was seeded into the 384-well cell culture plate. The plate was subsequently incubated at 37 °C in the dark for 120 min. Post-incubation, the cell plate was equilibrated to room temperature for 10–20 min, and 5 μL of the detection reagent were added to each well. Following a 60 min incubation at 25 °C, chemiluminescent readings were obtained using the envision system.

##### Cell Culture

The cells were maintained at 37 °C with 5% CO_2_ in McCoy’s 5a medium with supplements and cultivated utilizing precise techniques to ensure accurate, reproducible results. Cells were gently dissociated for assays to maintain receptor integrity and resuspended in Opti-MEM^®^ for compatibility with the PathHunter^®^ (DiscoverX, Shanghai, China). Detection Kit. Cell density was adjusted to 4000 cells per well in a 384-well plate, to ensure that a uniform monolayer was established for the β-galactosidase assay. After seeding and incubation in the dark at 37 °C to facilitate cell attachment, the plates were equilibrated to room temperature prior to compound treatment. Serial dilutions of the compounds were assessed for pharmacological effect. A detection reagent was then added to measure β-galactosidase activity as indicative of μ-opioid receptor and β-arrestin2 interaction. Following reagent incubation, chemiluminescence was quantified, providing quantitative data on compound agonistic activity. Throughout, meticulous aseptic technique and strict adherence to protocols were imperative to ensure the integrity of the pharmacological data derived from these studies.

### 3.6. In Vivo Pharmacological Experiments

#### 3.6.1. Acetic Acid-Induced Writhing Test

The acetic acid-induced writhing test was performed as described previously [24]. In brief, after pretreatment with the test compound at a dose of 20 mg/kg via intravenous tail vein injection, 1% (*v*/*v*) glacial acetic acid was administered intraperitoneally (i.p.) at a dose of 10 mL/kg. Subsequently, the number of writhing responses over a 20 min period was recorded [24]. The antinociception rate was calculated as the percentage of maximal possible effect (%MPE), where %MPE = 100 × (NWV − NWT)/NWV. NWV and NWT represent the average number of writhing responses of the vehicle and test groups, respectively.

#### 3.6.2. Hot-Water Tail Withdrawal Test

The hot-water tail withdrawal test was performed as described previously [24]. In brief, the hot-water tail withdrawal test was conducted using a water bath maintained at 55 ± 0.5 °C. The distal 3 cm section of the mouse’s tail was immersed perpendicularly into the hot water. The duration for which the tail remained in the water was recorded as the initial tail withdrawal latency (TWL0). Only untreated mice demonstrating an initial tail withdrawal latency (TWL0) of less than 7 s were utilized in the study. Following the administration of a single dose of the test compound (20 mg/kg, i.v.) or vehicle, the subsequent tail withdrawal latency (TWL) was measured at various time intervals (15, 30, 45, 60, and 90 min). To prevent potential tissue damage, a maximum cutoff latency of 16 s was enforced [24]. Antinociception was quantified using the percentage of maximal possible effect (%MPE), calculated as %MPE = 100 × (TWL − TWL0)/(16 − TWL0).

#### 3.6.3. Formalin Paw-Licking Test

The formalin injection assay was performed as described previously [24]. In brief, the vehicle (0.1 mL saline/10 g body weight, n = 8), the positive control of PZM21 (20 mg/kg, n = 8), or **6a**~**6l** (20 mg/kg, n = 8) were administered via tail vein injection. After 15 min, 30 µL of a 2.7% formalin solution was administered into the right hind paw of each mouse. Then, the mice were put into the cage and their behaviors were observed. The antinociception rate was quantified as the percentage of maximal possible effect (%MPE), calculated as %MPE = 100 × (PLTV − PLTT)/PLTV; PLTV and PLTT represent the average paw-licking duration during Phase II for the vehicle and test groups, respectively.

### 3.7. Physical Dependence and Respiratory Depression Assessments

#### 3.7.1. Physical Dependence

Groups of mice received intravenous (i.v.) administration of either saline or the drug at a dose of 20 mg/kg (with morphine administered at 4 mg/kg), once daily for 5 days, between 8 and 12 a.m. Two hours after the final intravenous (i.v.) administration on day 5, the mice were administered naloxone (2 mg/kg, intraperitoneally i.p.), and the number of vertical jumps was tallied over a 20 min duration to evaluate the physical dependence on the test compound [24,25].

#### 3.7.2. Respiratory Depression

All mice underwent a 12 h fast with ad libitum access to water before the experiments. Following a single intravenous (i.v.) administration of either saline or the drug at a dose of 20 mg/kg (with morphine at 4 mg/kg), blood samples were collected via retro-orbital puncture from the mice at several time intervals thereafter (5, 10, 15, 20, and 40 min), and partial oxygen pressure (pO_2_) levels were measured using an ABL90 FLEX blood gas analyzer (Radiometer, Copenhagen, Denmark) [24].

### 3.8. Docking Simulations

The (pdb: 7SBF) PDB files were downloaded from the PDB site (http://www.rcsb.org/, accessed on 21 August 2023). Then, to remove water molecules and add hydrogen atoms, Chain R of 7SBF was selected for subsequent molecular docking. The PDB docking grid was set to the agonist binding site for candidate compound (**6a**) docking. PDB files (7SBF chain R) were converted to the PDBQT format as macromolecules before molecular docking. The grid (ligand docking search space) was located as described above. Then, AutoDock Vina 1.1.2 [26] was used for the subsequent molecular docking. Protein–ligand interactions were visualized using PyMOL version 2.5.4. The amino acid residues of PDB(7SBF) close to the hit ligands (≤4 Å) were highlighted as potential interactive residues involved in the protein–ligand interaction.

### 3.9. Statistical Analysis

Prism 8.0.2 (GraphPad Software, San Diego, CA, USA) was employed for data management and statistical analysis. The results are presented as the mean ± standard error of the mean (SEM). The EC_50_ and Emax values were calculated using nonlinear regression analysis, specifically a three-parameter model. Significant changes resulting from the application of the substance were evaluated using a two-tailed Student’s *t* test, with baseline activity serving as the common control. A *p*-value of <0.05 was considered to denote statistical significance.

## 4. Conclusions

In conclusion, a set of new PZM21 derivatives as µOR-biased agonists was synthesized, and their G_i/o_-protein signaling potency and antinociceptive efficacy were evaluated. This study confirms that the novel PZM21 compounds maintained essential functional groups even though their structure had been altered, resulting in potent µOR agonists with reduced β-arrestin recruitment. New compound **6a** demonstrated strong antinociceptive effects in various pain models, with reduced adverse effects such as respiratory depression and physical dependence compared to the common opioid morphine, indicating a potentially safer profile. Additionally, the efficient binding of **6a** to µOR, as revealed through docking studies, underscores its potential as a lead compound in the development of safer opioids for pain management.

## Figures and Tables

**Table 1:** The EC_50_ values and the maximum responses of the tested compounds at the µOR-G_i/o_ activation assay.

Compound	EC_50_ (nM) (95% CI)	Max Effect ± SEM (%)
DAMGO	2.196 (1.955–3.361)	100.85 ± 0.56 (2 μM)
Morphine	21.83 (15.07–31.61)	100.99 ± 2.03 (10 μM)
PZM21	1.615 (1.302–2.002)	89.75 ± 2.93 (10 μM)
**6a**	10.82 (4.381–26.77)	51.86 ± 1.06 (500 nM)
**6b**	109.6 (68.77–174.7)	73.91 ± 2.38 (50 μM)
**6c**	72.83 (30.81–130.0)	42.67 ± 0.27 (500 nM)
**6d**	87.98 (65.80–117.6)	87.85 ± 1.18 (10 μM)
**6e**	-	−1.41 ± 5.21 (500 nM)
**6f**	106.6 (83.14–136.6)	84.82 ± 1.86 (10 μM)
**6g**	59.49 (25.48–138.9)	24.64 ± 0.40 (1 μM)
**6h**	13.12 (4.196–41.01)	41.70 ± 0.07 (10 μM)
**6i**	-	7.10 ± 0.77 (500 nM)
**6j**	97.83 (39.61–241.61)	27.20 ± 1.71 (10 μM)
**6k**	-	20.35 ± 10.61 (100 pM)
**6l**	-	18.09 ± 1.81 (1 nM)

DAMGO, [D-Ala2, N-MePhe4, Gly-ol]-enkephalin; EC_50_, half-maximal effective concentration; -, no measurable activity. The data presented here encapsulate the mean values, accompanied by either the standard error of the mean (SEM) or the 95% confidence intervals across ten different concentrations, each replicated three times (*n* = 3).

**Table 2:** The EC_50_ values and the maximum responses of the tested compounds at the µOR β-arrestin recruitment assay.

Compound	EC_50_ (nM) (95% CI)	Max Effect ± SEM (%)
DAMGO	100.6 (86.13–153.8)	91.19 ± 5.50 (3 μM)
Morphine	621.5 (438.7–880.4)	21.41 ± 4.00 (3 μM)
PZM21	36.96 (14.28–95.63)	3.09 ± 0.42 (1 μM)
**6a**	-	0.38 ± 0.21 (100 μM)
**6b**	-	1.99 ± 1.77(100 μM)
**6c**	-	1.06 ± 0.93 (50 μM)
**6d**	-	7.72 ± 2.88 (100 μM)
**6e**	-	2.95 ± 1.37 (100 μM)
**6f**	-	5.45 ± 1.75 (100 μM)
**6g**	-	1.12 ± 0.52 (50 μM)
**6h**	-	2.19 ± 1.91 (100 μM)
**6i**	-	1.96 ± 0.72 (100 μM)
**6j**	-	1.63 ± 0.32 (50 μM)
**6k**	-	4.15 ± 0.02 (50 μM)
**6l**	-	1.96 ± 0.45 (50 μM)

DAMGO, [D-Ala2, N-MePhe4, Gly-ol]-enkephalin; EC_50_, half-maximal effective concentration; -, no measurable activity. The data presented here encapsulate the mean values, accompanied by either the standard error of the mean (SEM) or the 95% confidence intervals across ten different concentrations, each replicated three times (*n* = 3).

## Data Availability

Data are contained within the article and Supplementary Materials.

## Supplementary Materials

The following supporting information can be downloaded at https://www.mdpi.com/article/10.3390/molecules29132961/s1: the synthesis route of compounds **e1**~**e12**, HPLC purity, HR-ESI-MS, ^1^H NMR, and ^13^C NMR spectra.
