# Supplementary material for: Synthesis and Biological Evaluation of Novel Biased Mu-Opioid Receptor Agonists
Source: Molecules. 2024 Jun 21;29(13):2961. doi: 10.3390/molecules29132961 (PMC11243066; doi:10.3390/molecules29132961)
Supplement: Supplementary file 1 [file molecules-29-02961-s001.zip › molecules-3047966-supplementary.pdf]

Supplementary Information

Synthesis and Biological Evaluation of Novel Biased Mu-Opioid Receptor Agonists

Yanhao Guo [1], Ruimin Yu [2], Tao Zhang [3], Fengxia Ren [3], Zixing Yu [3], Jingchao Cheng [3], Hongxin Jia [3], Weiguo Shi [3,*] and Yatong Zhang [1,*]

1 College of Science, Hebei University of Science and Technology, Shijiazhuang 050018, China; guoyanhao123@126.com
2 Center for Disease Control and Prevention of Central Theater Command, PLA, No.66 Heishitou Road, Beijing 100042, China; yrm0601@163.com
3 Beijing Institute of Pharmacology & Toxicology, 27 Tai-Ping Road, Beijing 100850, China; ren2019victory@126.com (F.R.)
* Correspondence: shiweiguo@bmi.ac.cn (W.S.); zhangyatong@hebust.edu.cn (Y.Z.); Tel: +010-66931639 (W.S.)

Synthesis of compounds **e1~e12**.

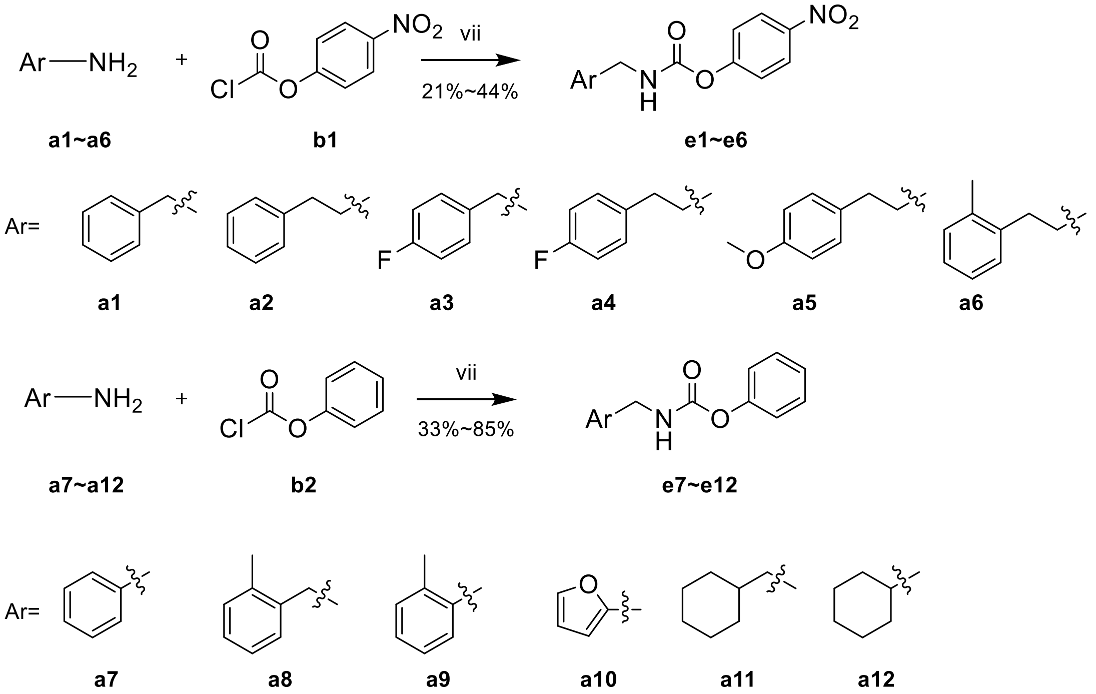

Scheme S1. Synthesis of compounds **e1~e12**. Reagents and Conditions: (vii) Et_3N, THF, ice bath then r.t., 8 h.

HPLC, HR-ESI-MS, ^{1}H and ^{13}C NMR spectra

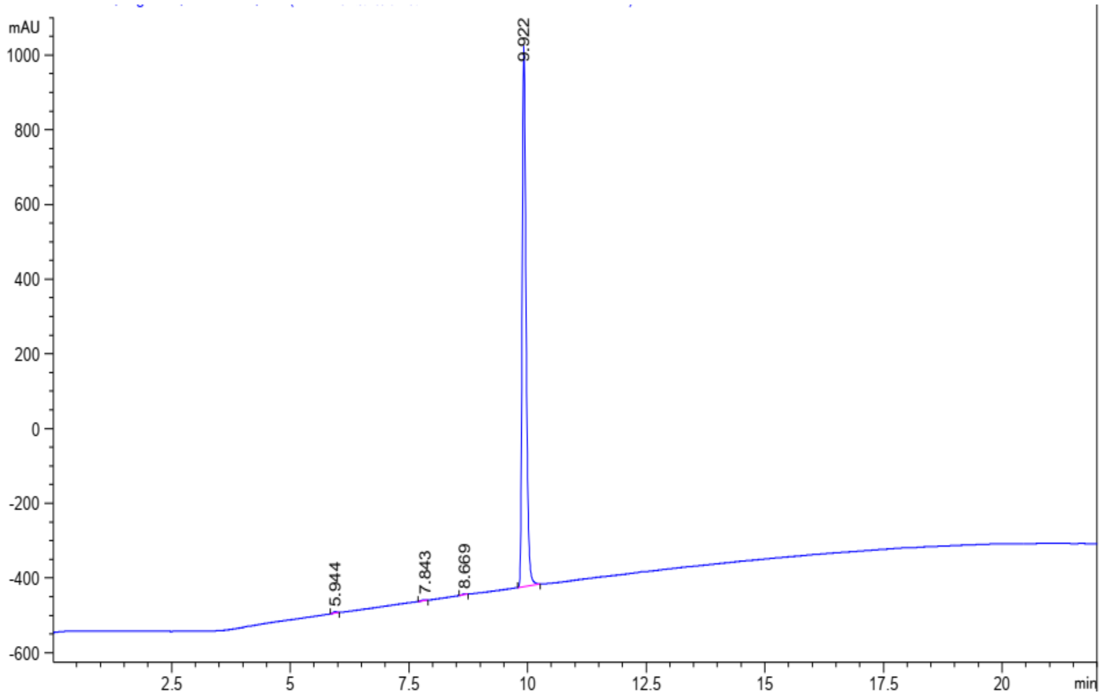

Figure S1 HPLC of compound **6a**

Qualitative Analysis Report

**Data Filename**	6132.d	**Sample Name**	3-P29-20221024-vi2-1
**Instrument Name**	TOF G6230A	**Acquired Time**	2022-11-04
**Acq Method**	YCL.M	**Acquired SW**	6200 series TOF/6500 series
**IRM Calibration Status**	Success		
**User Chromatograms**			

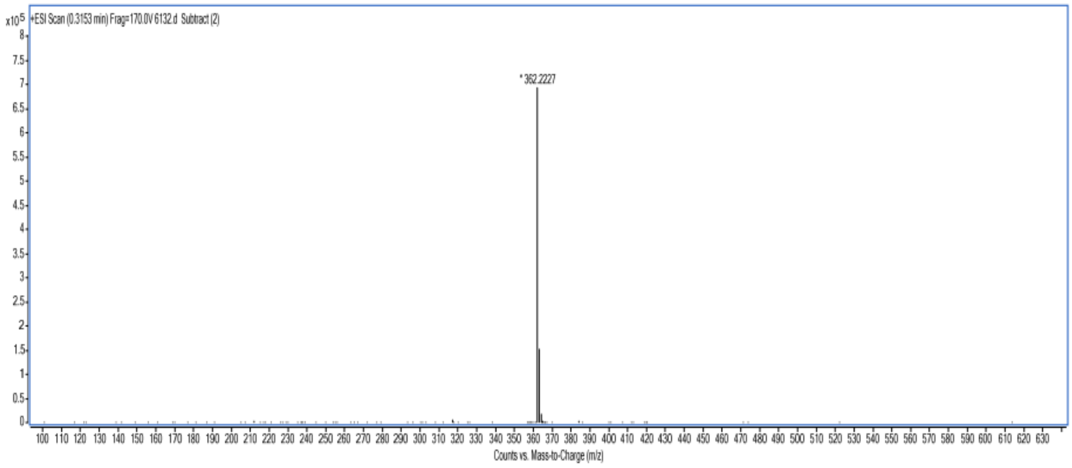

Figure S2 HR-ESI-MS of compound **6a**

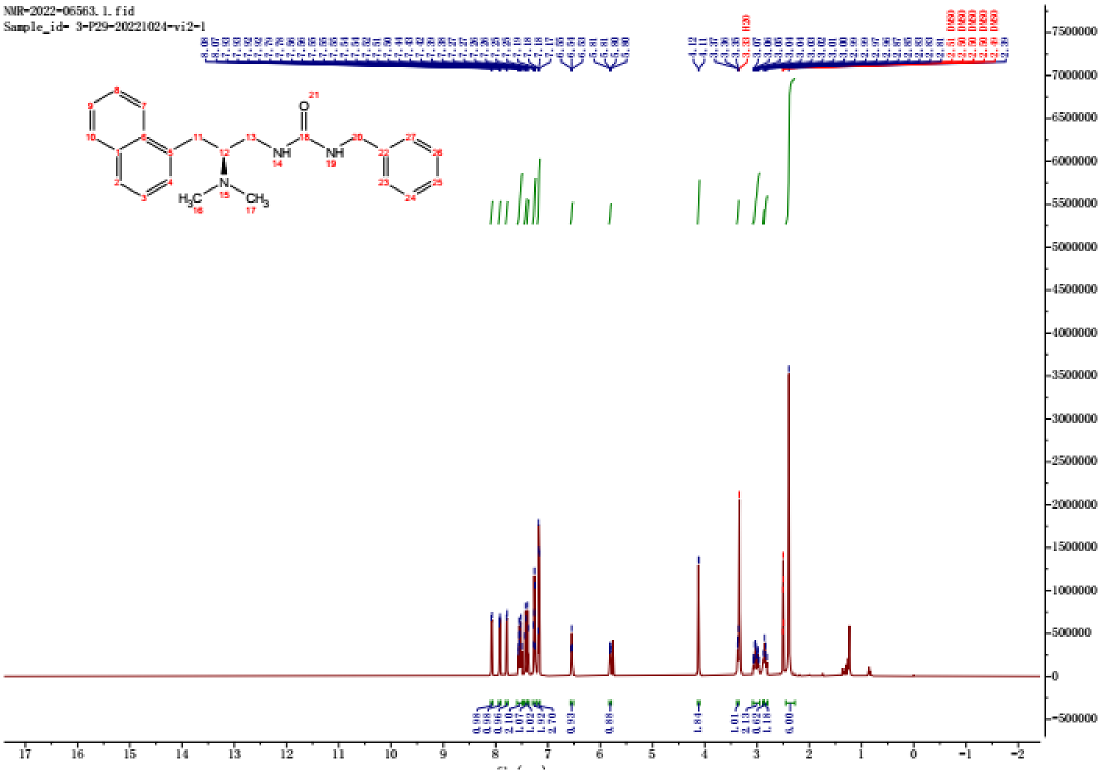

Figure S3 ^{1}H NMR spectra of compound **6a** in DMSO-d_6

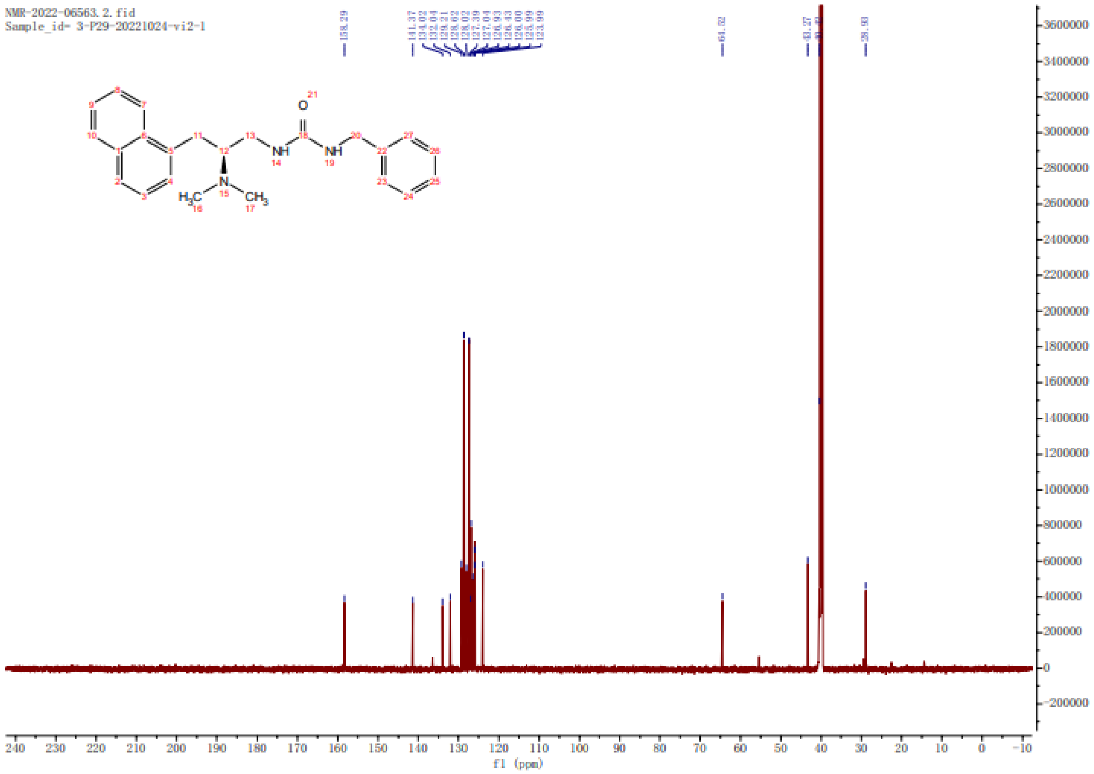

Figure S4 ^{13}C NMR spectra of compound **6a** in DMSO-d_6

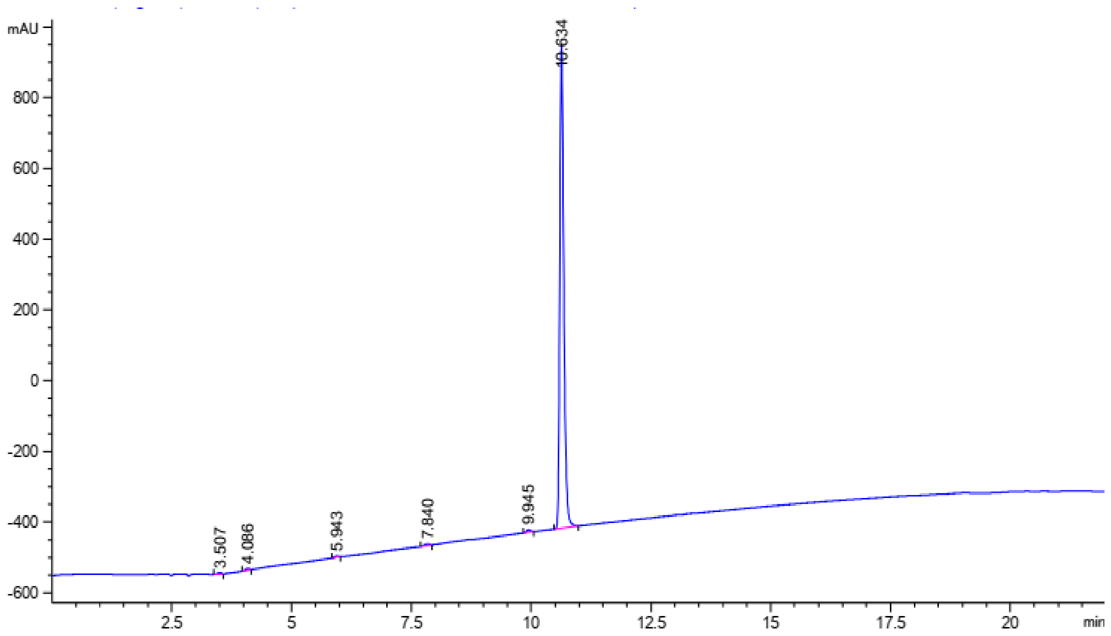

Figure S5 HPLC of compound **6b**

Qualitative Analysis Report

**Data Filename**	6133.d	**Sample Name**	3-P30-20221028-vi3-1
**Instrument Name**	TOF G6230A	**Acquired Time**	2022-11-04
**Acq Method**	YCL.M	**Acquired SW**	6200 series TOF/6500 series
**IRM Calibration Status**	**Success**		
**User Chormatograms**			

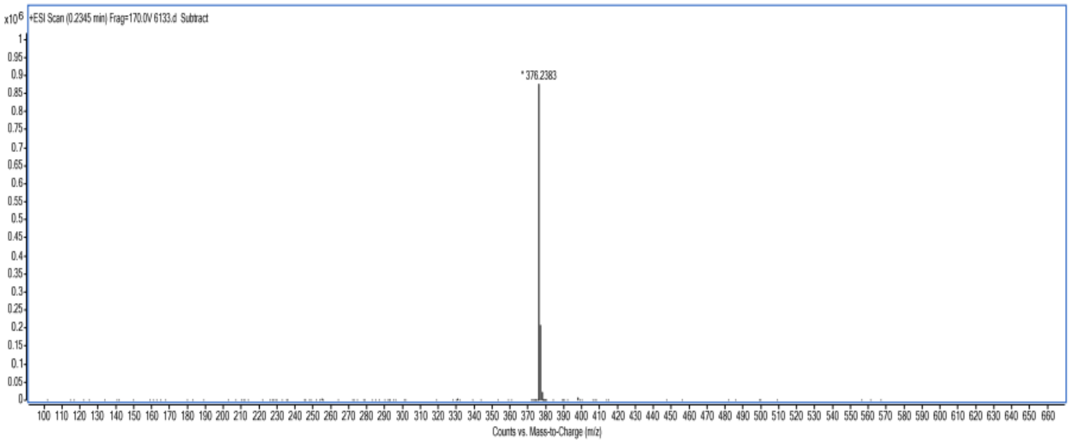

Figure S6 HR-ESI-MS of compound **6b**

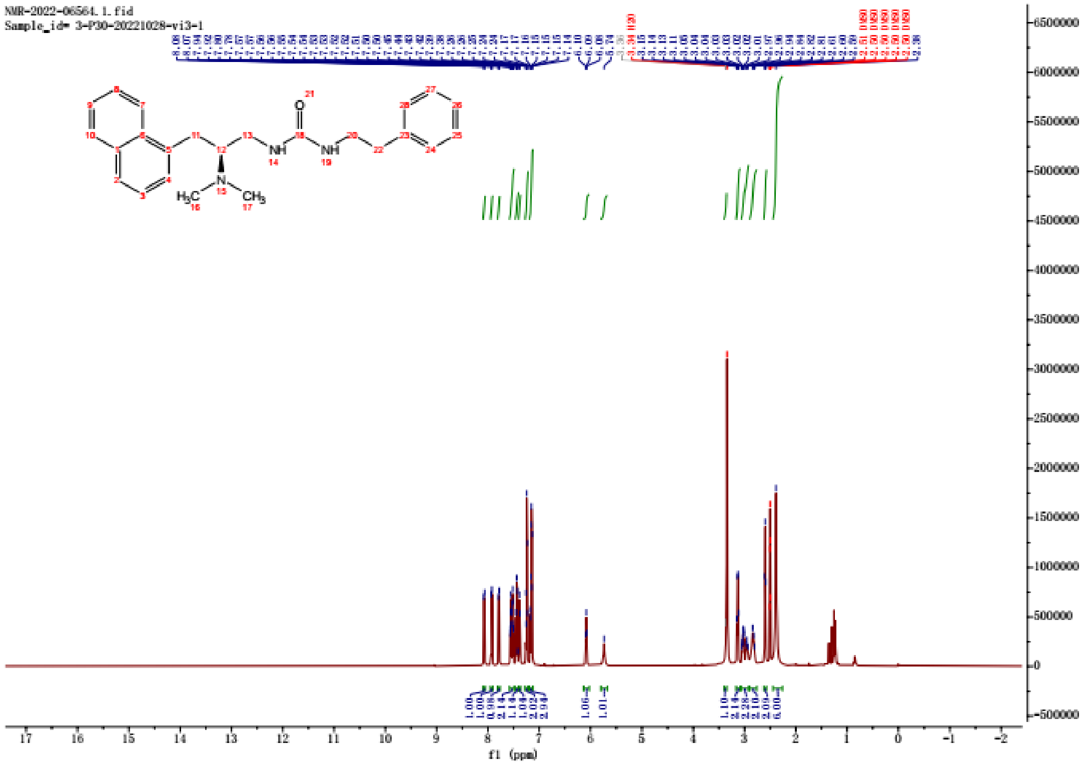

Figure S7 ^{1}H NMR spectra of compound **6b** in DMSO-d_6

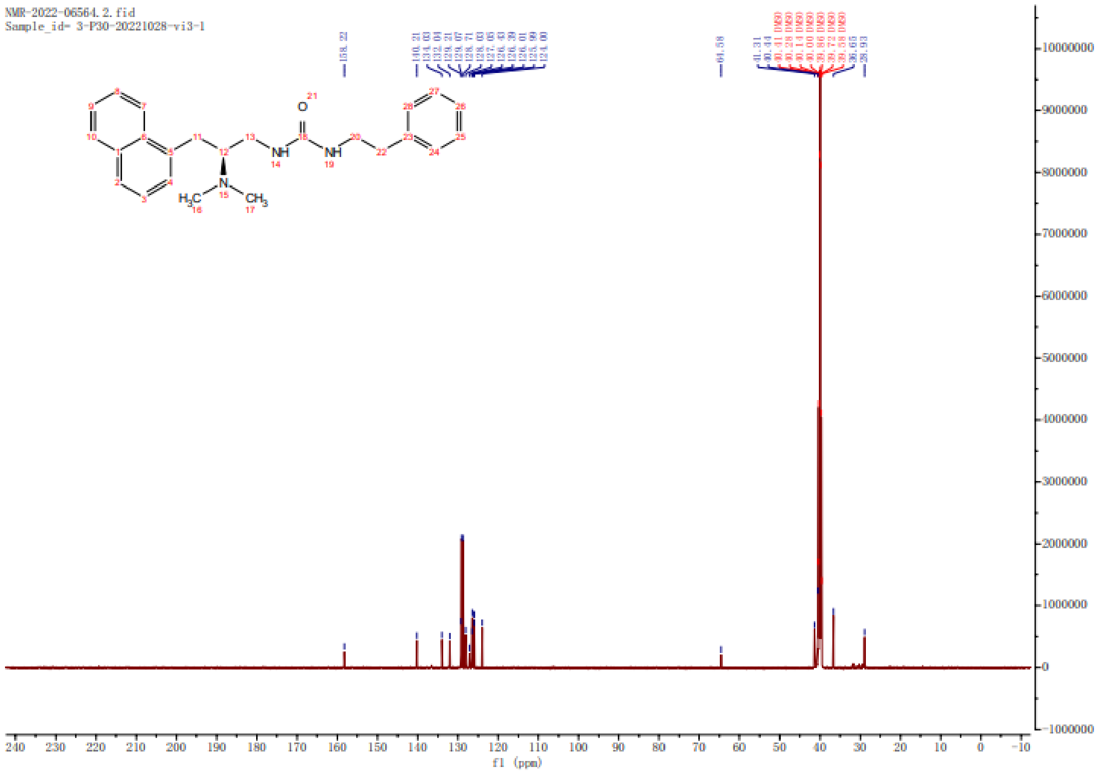

Figure S8 ^{13}C NMR spectra of compound **6b** in DMSO-d_6

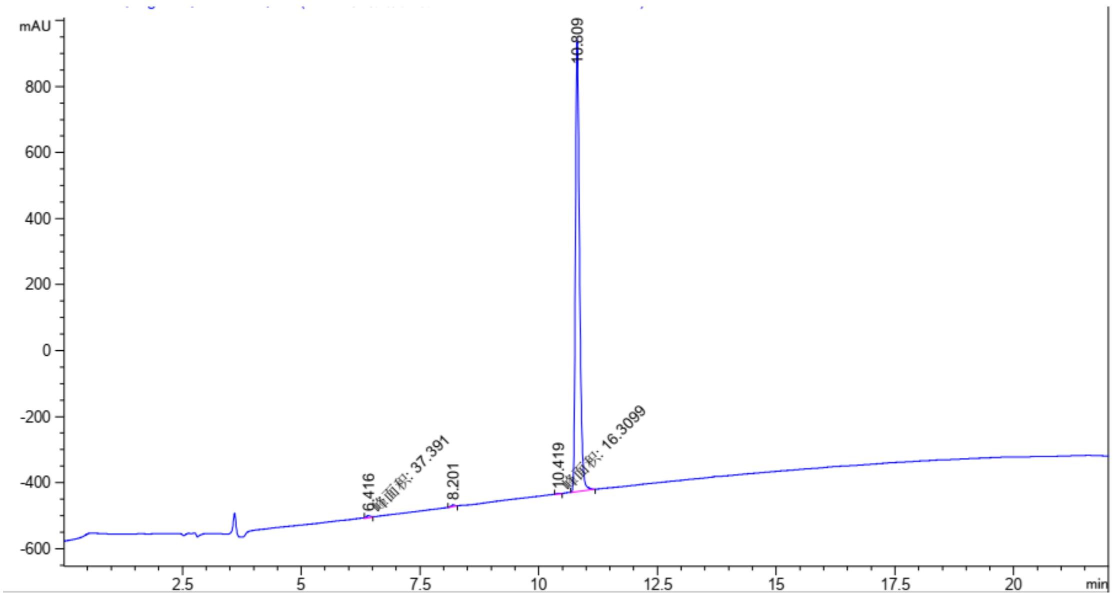

Figure S9 HPLC of compound **6c**

Qualitative Analysis Report

**Data Filename**	6518.d	**Sample Name**	3-P33-20221107-vi4-1
**Instrument Name**	TOF G6230A	**Acquired Time**	2022-12-01
**Acq Method**	YCL.M	**Acquired SW**	6200 series TOF/6500 series
**IRM Calibration Status**	Success		

User Chormatograms

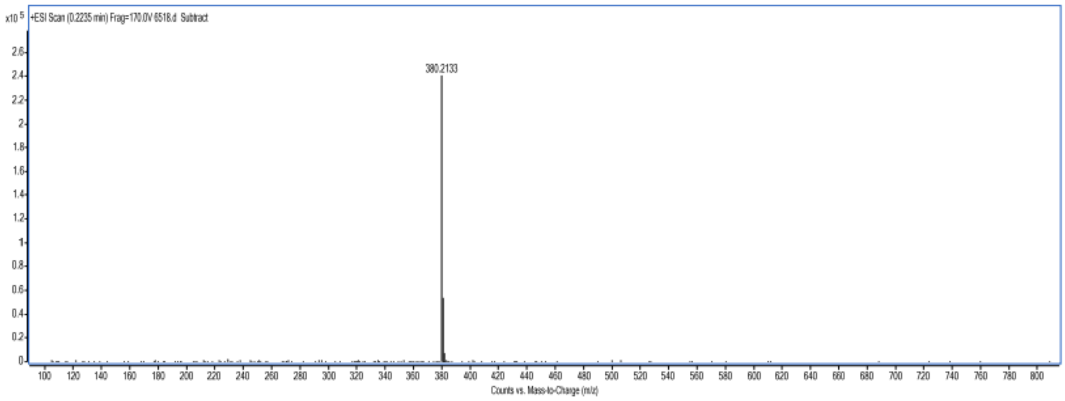

Figure S10 HR-ESI-MS of compound **6c**

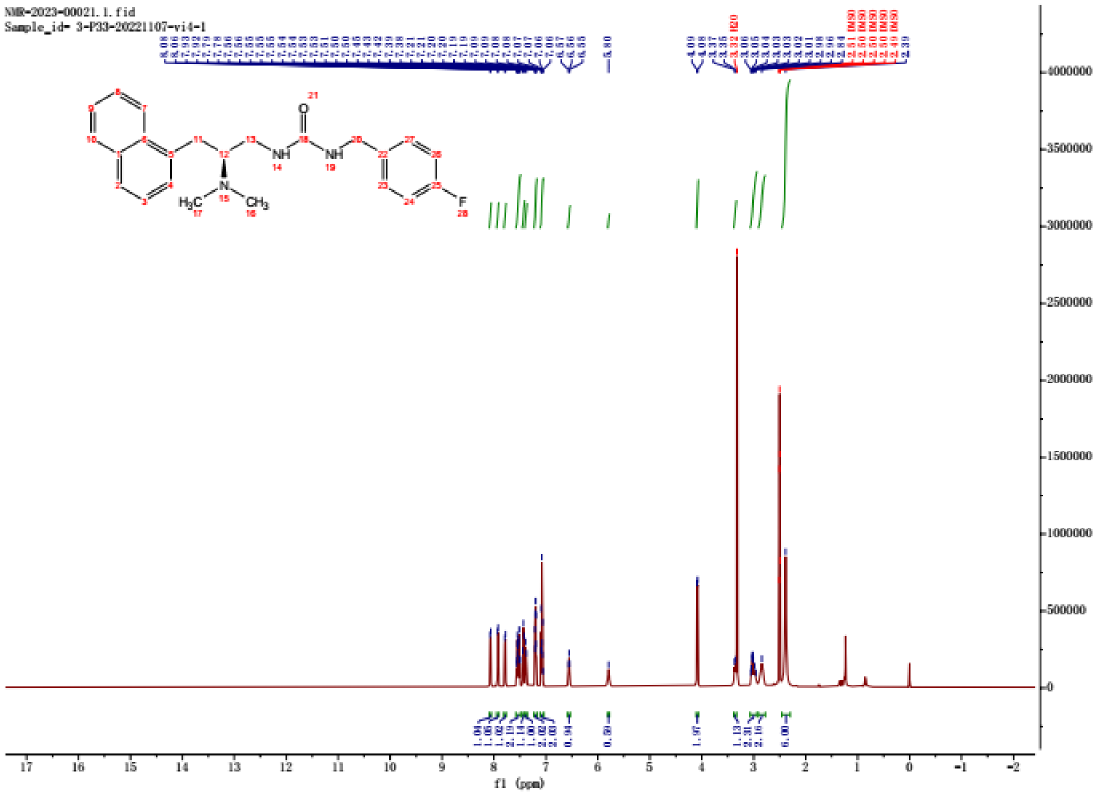

Figure S11 ^{1}H NMR spectra of compound **6c** in DMSO-d_6

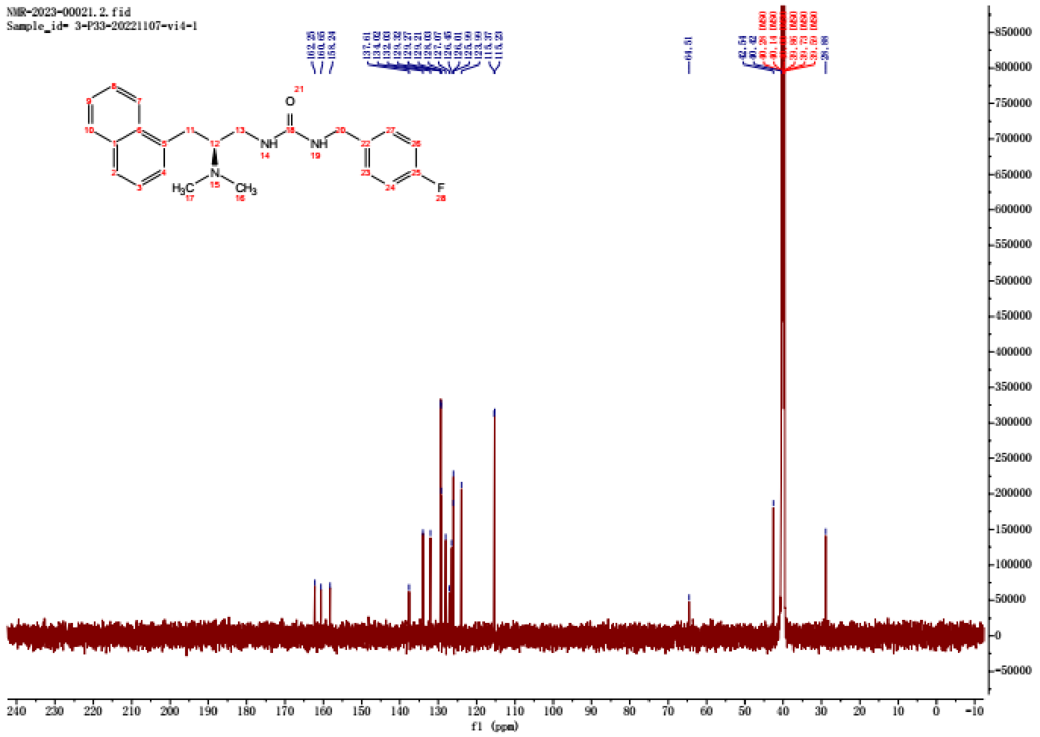

Figure S12 ^{13}C NMR spectra of compound **6c** in DMSO-d_6

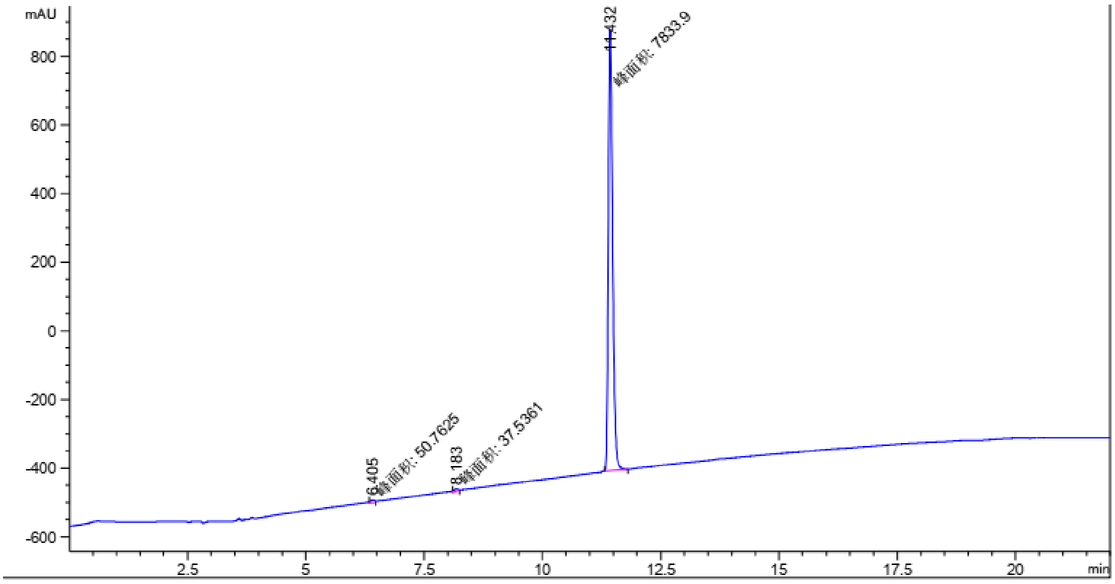

Figure S13 HPLC of compound **6d**

Qualitative Analysis Report

**Data Filename**	6520.d	**Sample Name**	3-P35-20221108-vi5-1
**Instrument Name**	TOF G6230A	**Acquired Time**	2022-12-01
**Acq Method**	YCL.M	**Acquired SW**	6200 series TOF/6500 series
**IRM Calibration Status**	Success		
**User Chormatograms**			

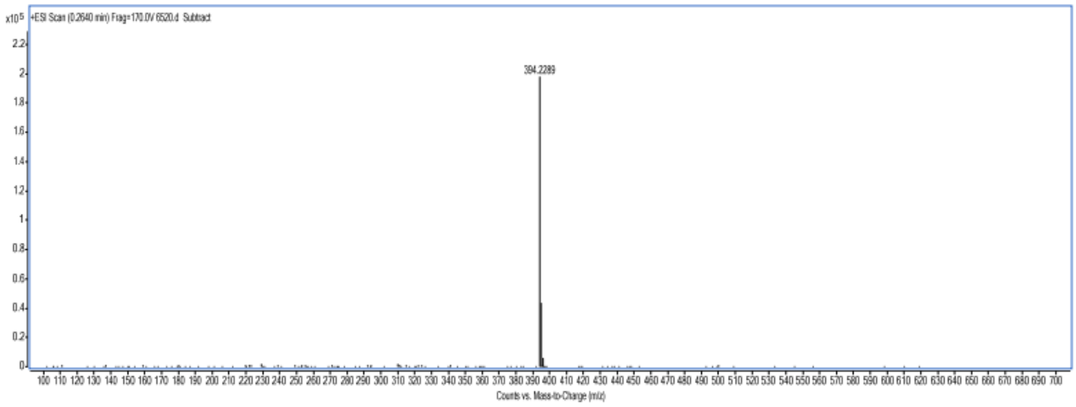

Figure S14 HR-ESI-MS of compound **6d**

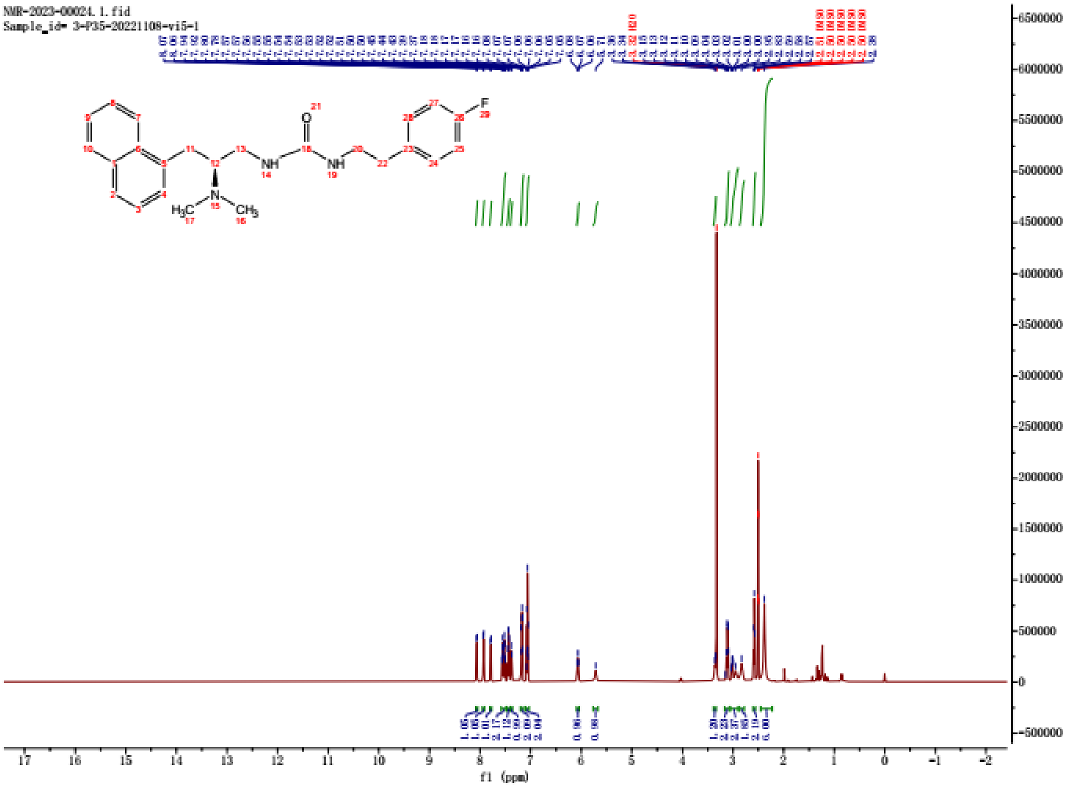

Figure S15 ^{1}H NMR spectra of compound **6d** in DMSO-d_6

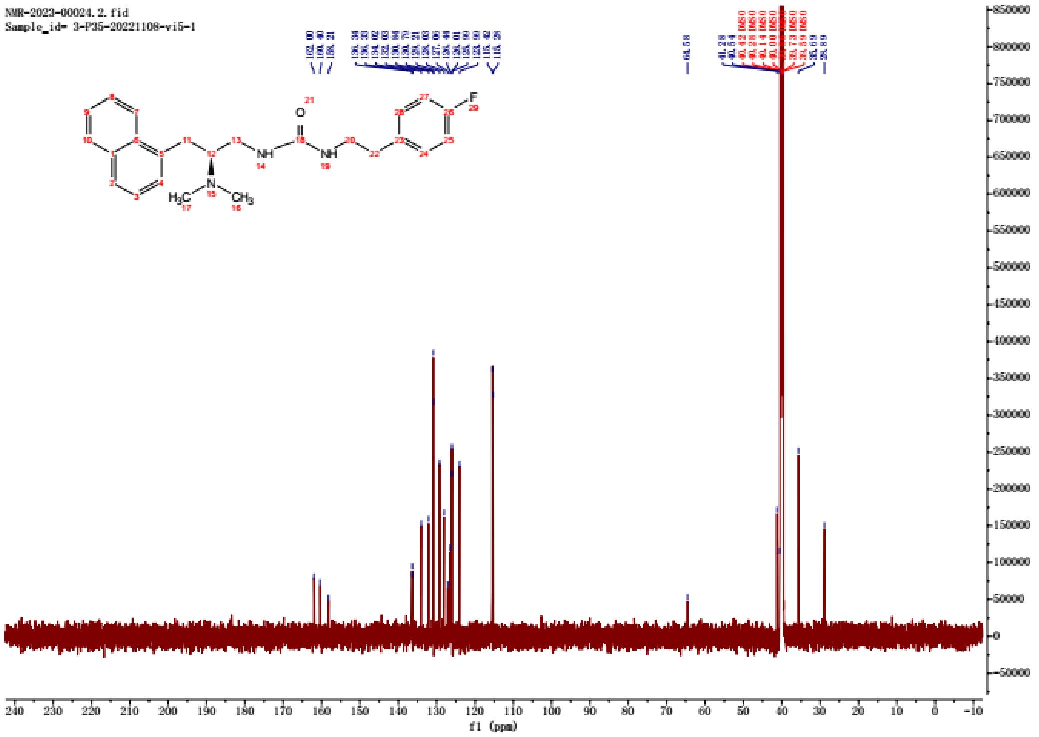

Figure S16 ^{13}C NMR spectra of compound **6d** in DMSO-d_6

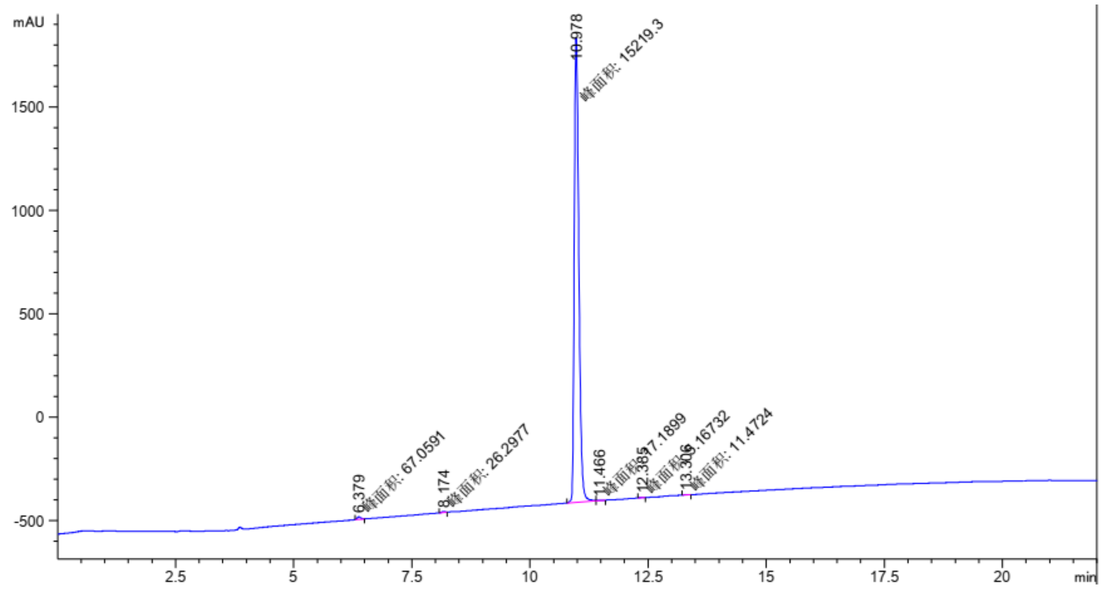

Figure S17 HPLC of compound **6e**

Qualitative Analysis Report

**Data Filename**	6523.d	**Sample Name**	3-P42-20221121-vi6-1
**Instrument Name**	TOF G6230A	**Acquired Time**	2022-12-01
**Acq Method**	YCL.M	**Acquired SW**	6200 series TOF/6500 series
**IRM Calibration Status**	Success		

User Chormatograms

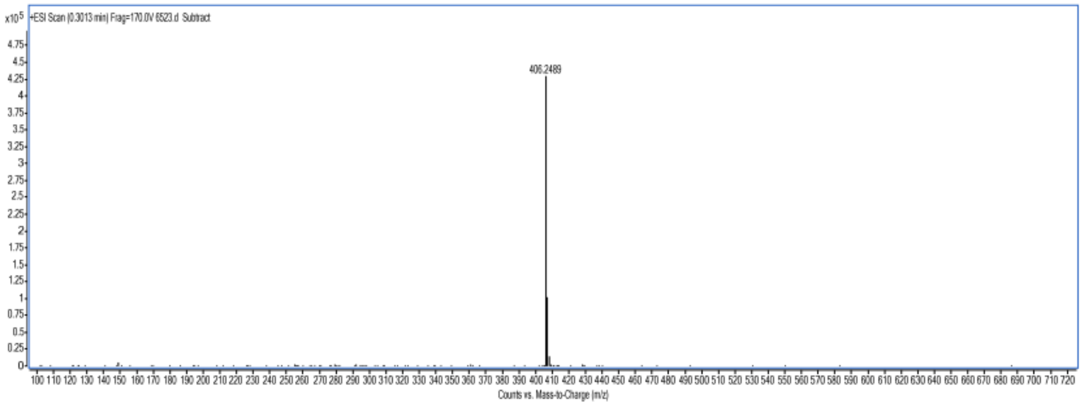

Figure S18 HR-ESI-MS of compound **6e**

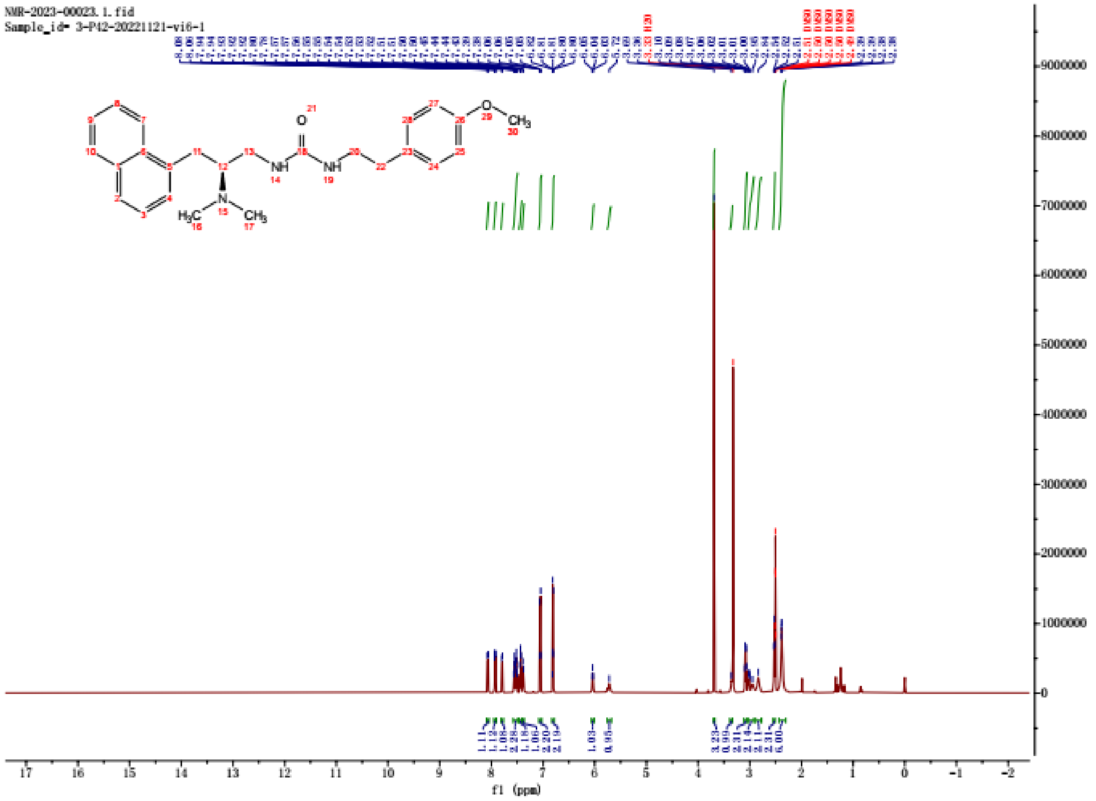

Figure S19 ^{1}H NMR spectra of compound **6e** in DMSO-d_6

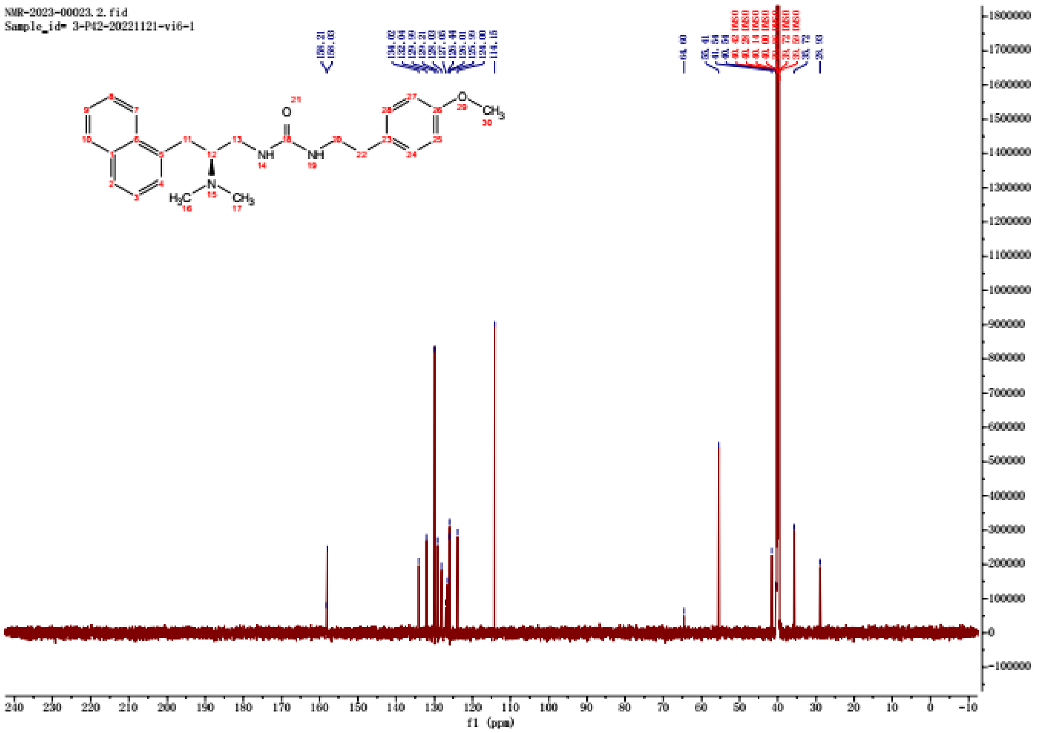

Figure S20 ^{13}C NMR spectra of compound **6e** in DMSO-d_6

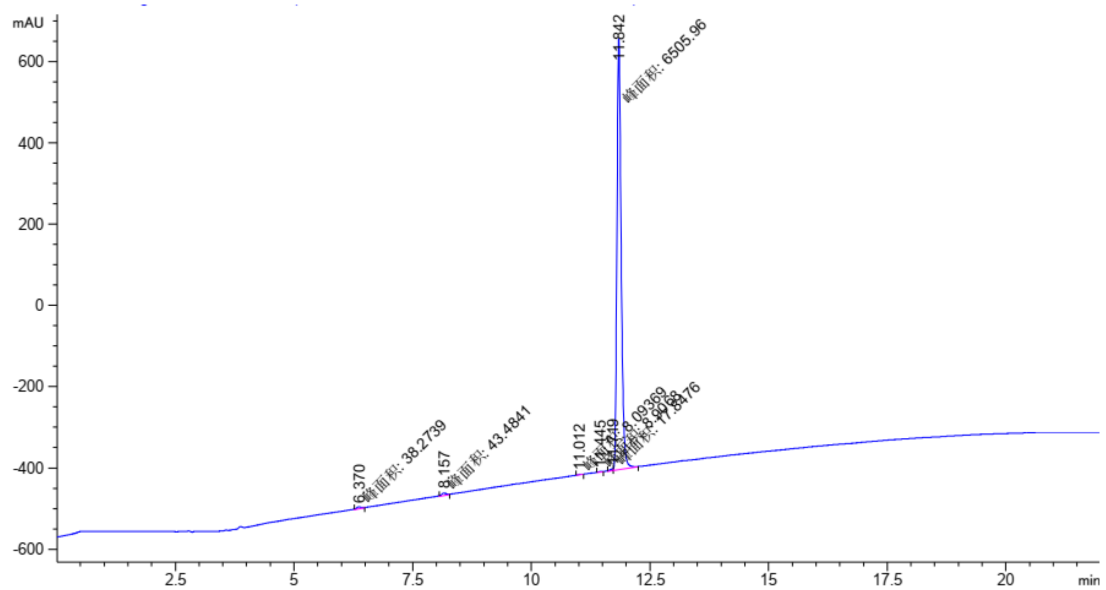

Figure S21 HPLC of compound **6f**

Qualitative Analysis Report

**Data Filename**	6524.d	**Sample Name**	3-P44-20221125-vi7-1
**Instrument Name**	TOF G6230A	**Acquired Time**	2022-12-01
**Acq Method**	YCL.M	**Acquired SW**	6200 series TOF/6500 series
**IRM Calibration Status**	Success		
**User Chormatograms**			

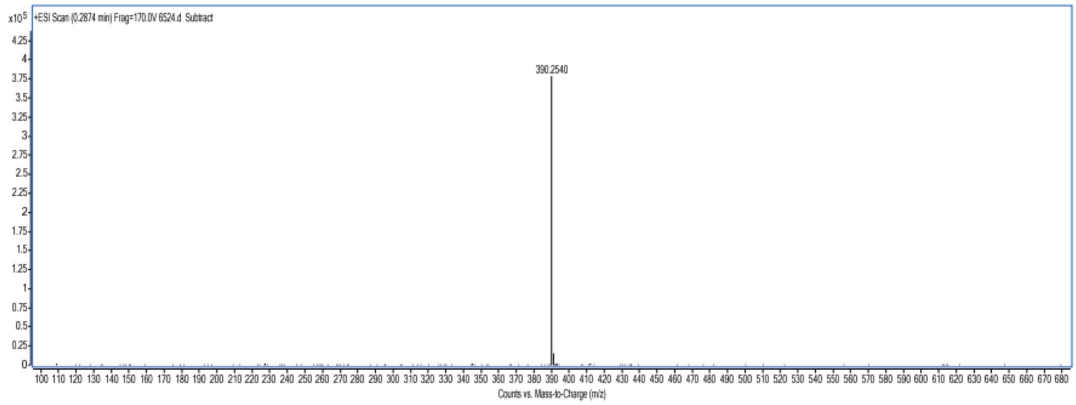

Figure S22 HR-ESI-MS of compound **6f**

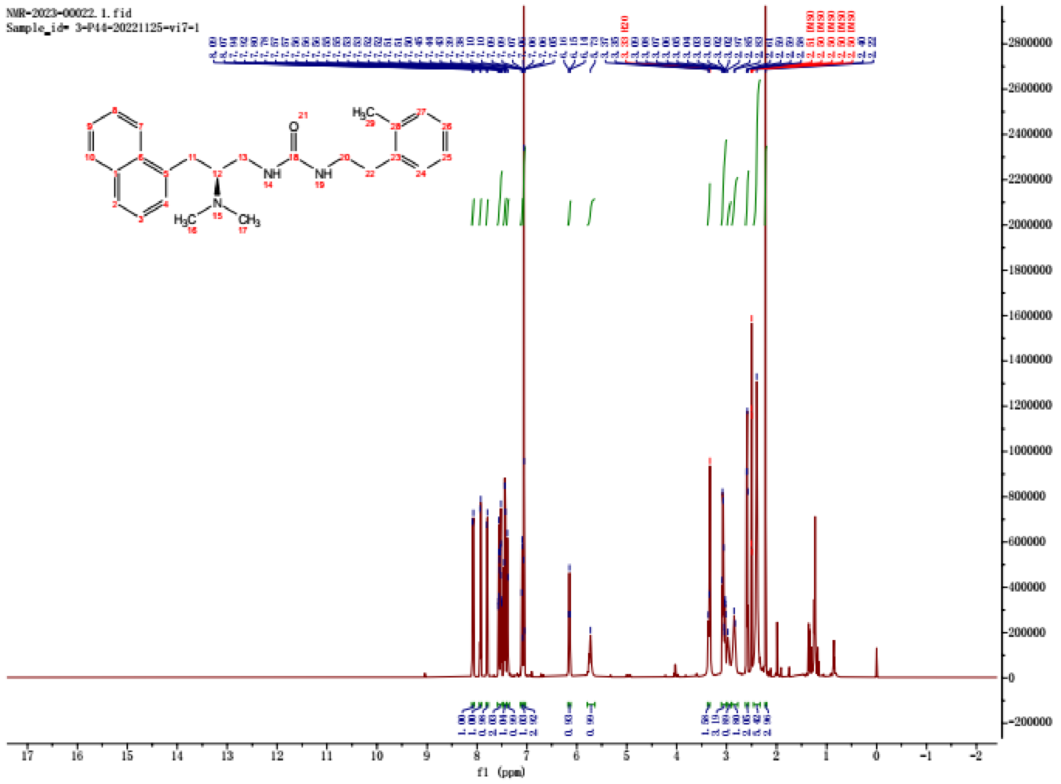

Figure S23 ^{1}H NMR spectra of compound **6f** in DMSO-d_6

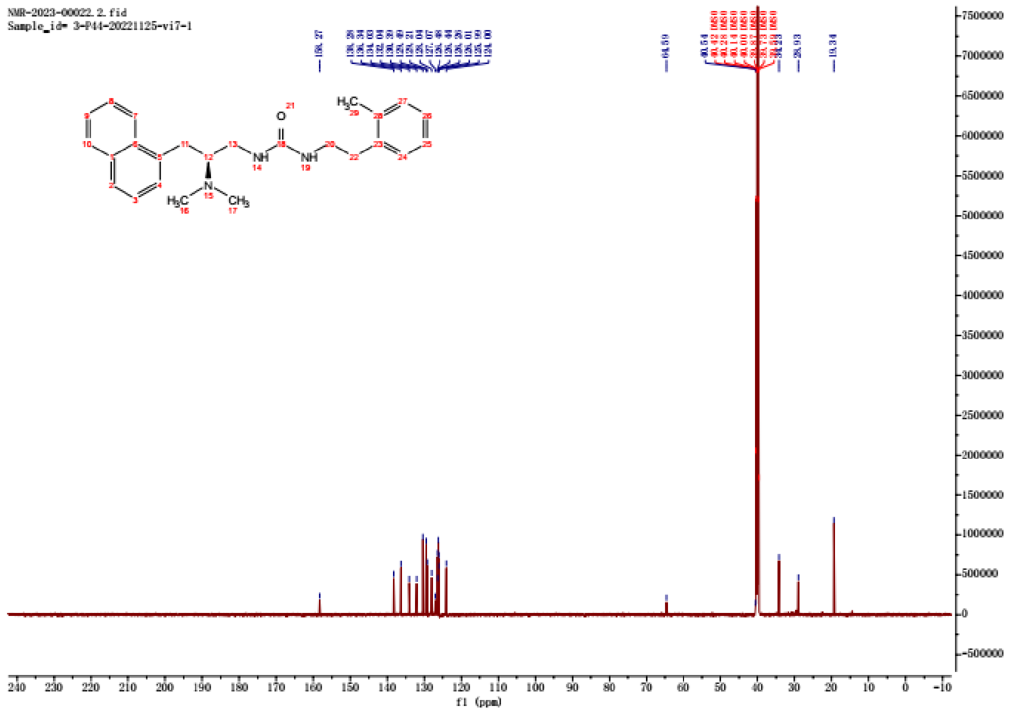

Figure S24 ^{13}C NMR spectra of compound **6f** in DMSO-d_6

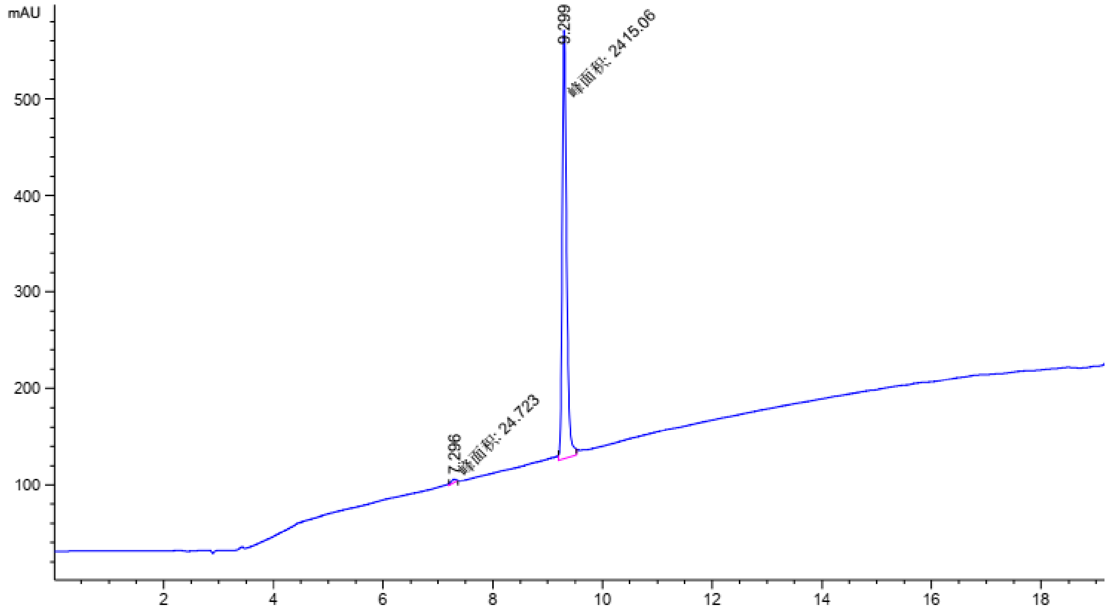

Figure S25 HPLC of compound **6g**

Qualitative Analysis Report

**Data Filename**	4729.d	**Sample Name**	5-P15-20230627-vi8-1
**Instrument Name**	TOF G6230A	**Acquired Time**	2023-07-20
**Acq Method**	YCL.M	**Acquired SW**	6200 series TOF/6500 series
**IRM Calibration Status**	Success		
**User Chormatograms**			

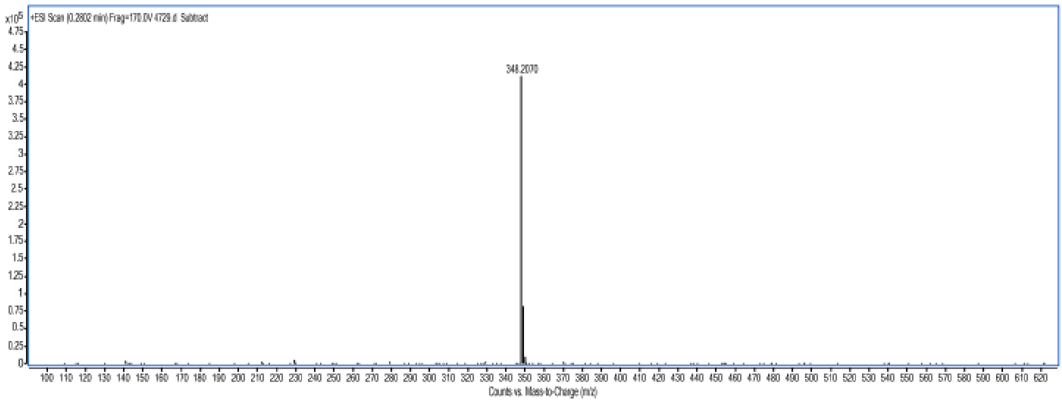

Figure S26 HR-ESI-MS of compound **6g**

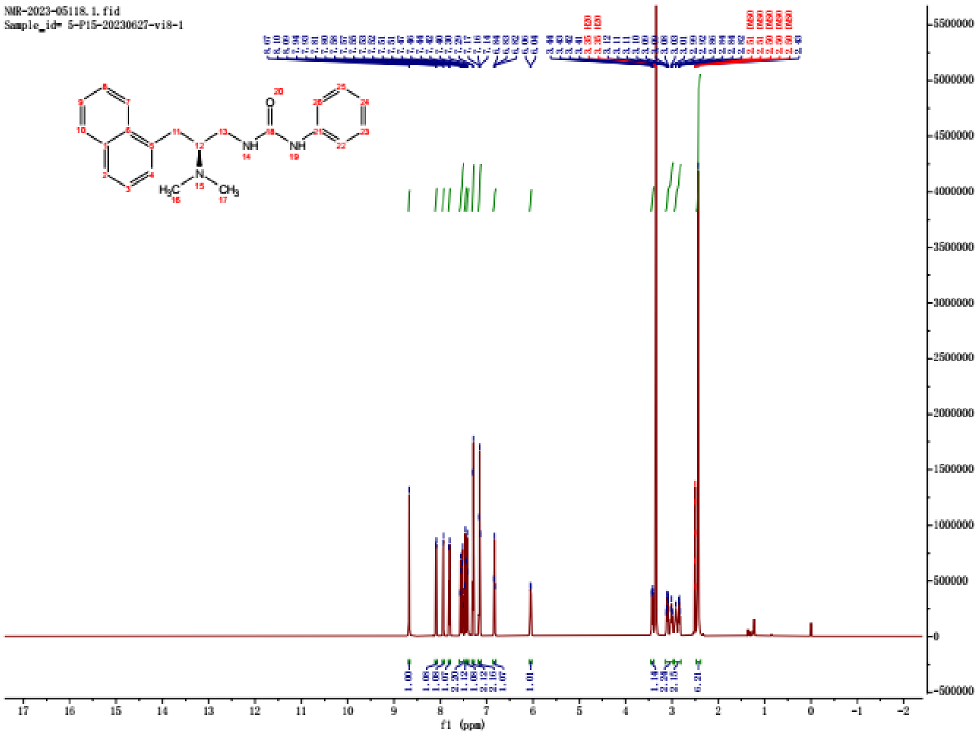

Figure S27 ^{1}H NMR spectra of compound **6g** in DMSO-d_6

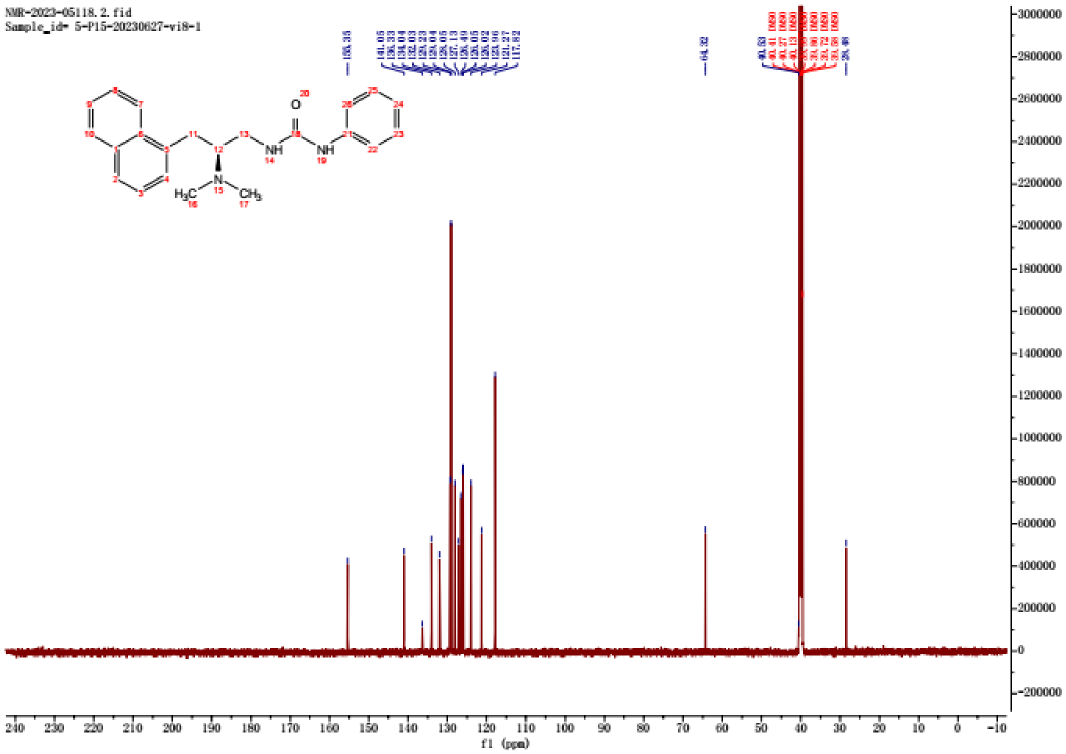

Figure S28 ^{13}C NMR spectra of compound **6g** in DMSO-d_6

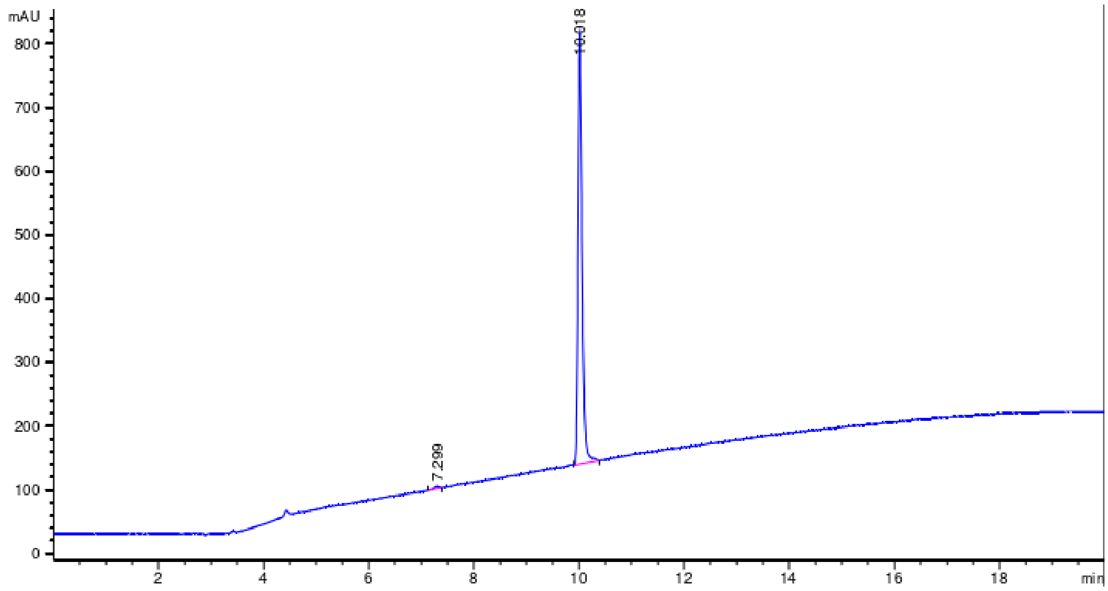

Figure S29 HPLC of compound **6h**

Qualitative Analysis Report

**Data Filename**	4730.d	**Sample Name**	5-P16-20230628-vi9-1
**Instrument Name**	TOF G6230A	**Acquired Time**	2023-07-20
**Acq Method**	YCL.M	**Acquired SW**	6200 series TOF/6500 series
**IRM Calibration Status**	Success		
**User Chormatograms**			

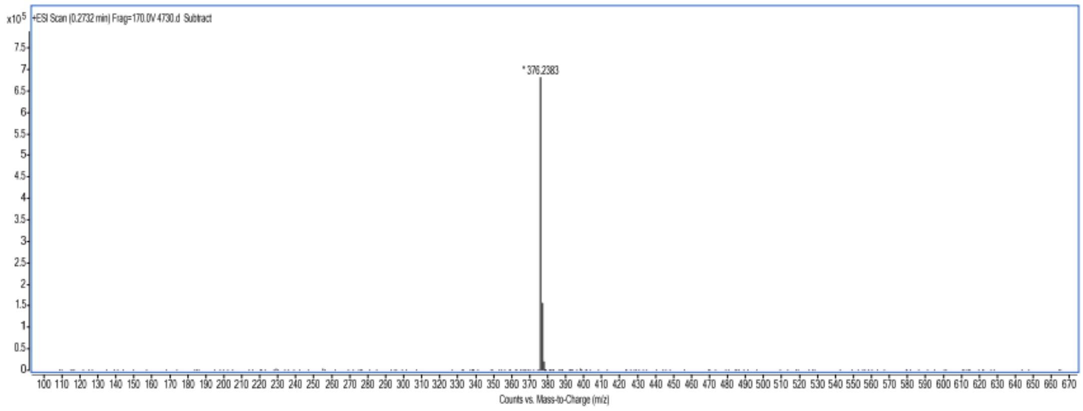

Figure S30 HR-ESI-MS of compound **6h**

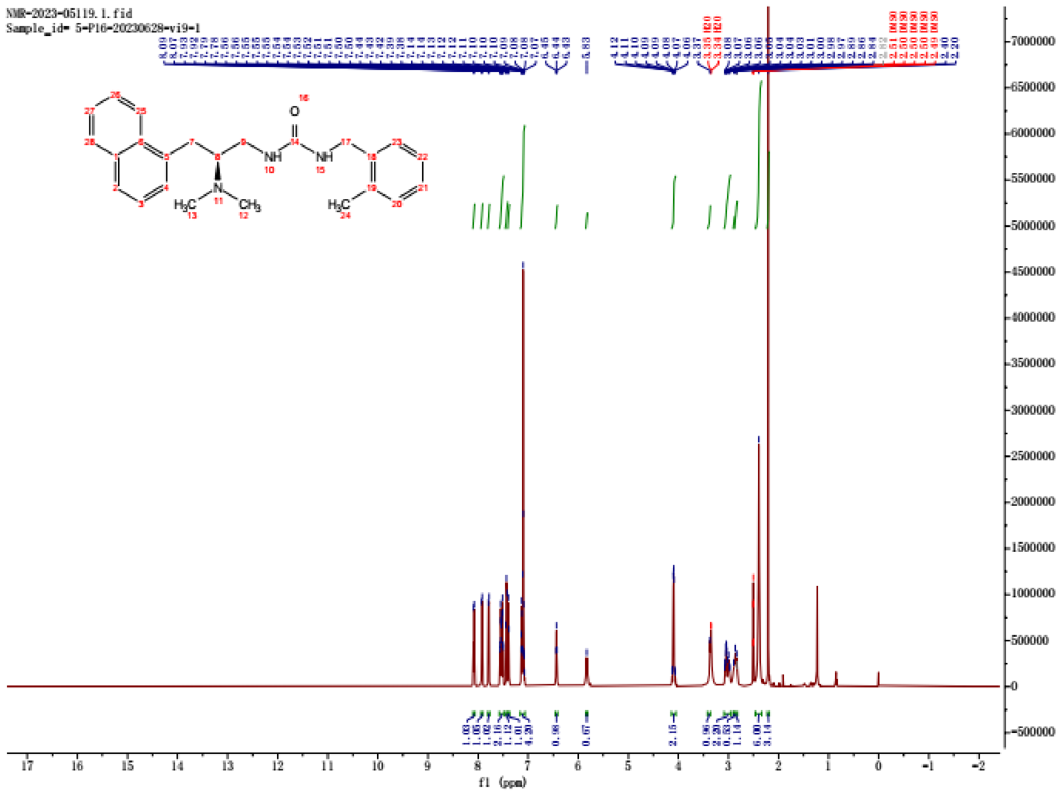

Figure S31 ^{1}H NMR spectra of compound **6h** in DMSO-d_6

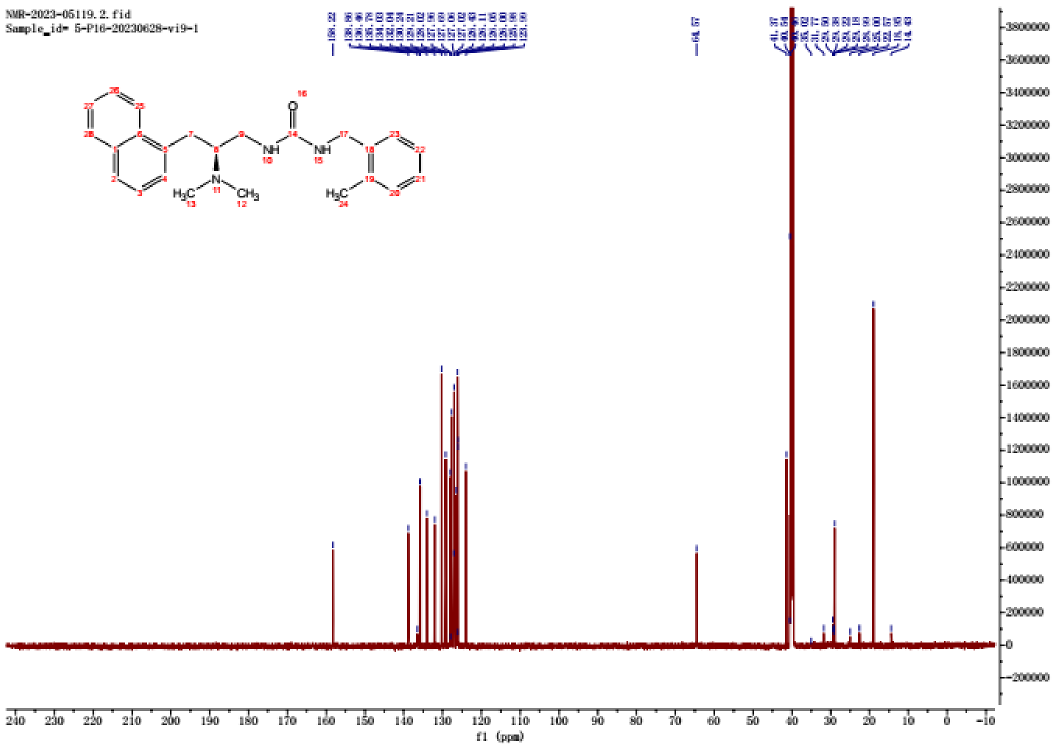

Figure S32 ^{13}C NMR spectra of compound **6h** in DMSO-d_6

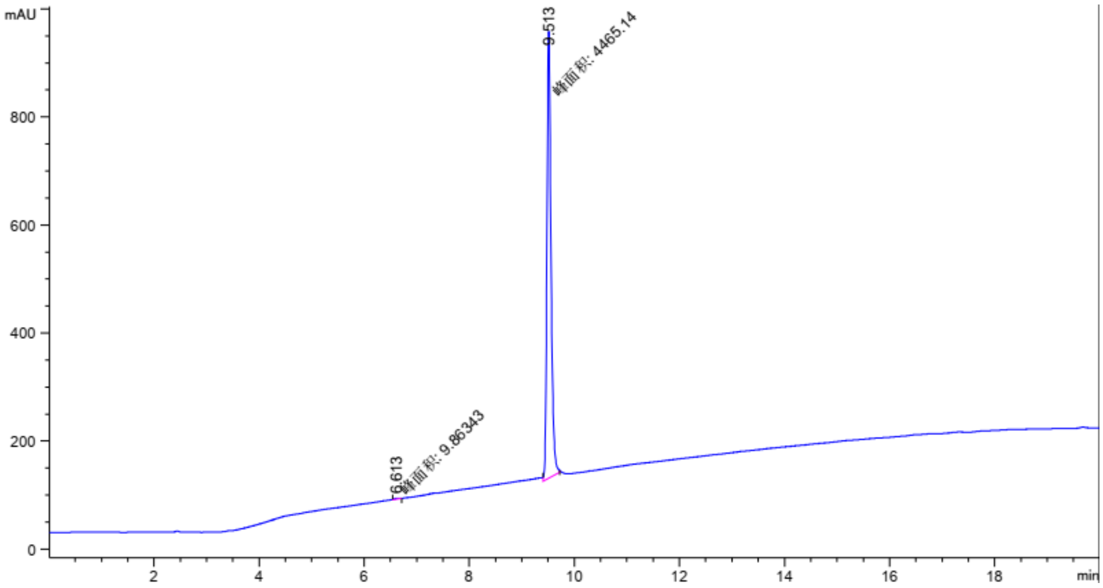

Figure S33 HPLC of compound **6i**

Qualitative Analysis Report

**Data Filename**	4731.d	**Sample Name**	5-P17-20230629-vi10-1
**Instrument Name**	TOF G6230A	**Acquired Time**	2023-07-20
**Acq Method**	YCL.M	**Acquired SW**	6200 series TOF/6500 series
**IRM Calibration Status**	Success		
**User Chormatograms**			

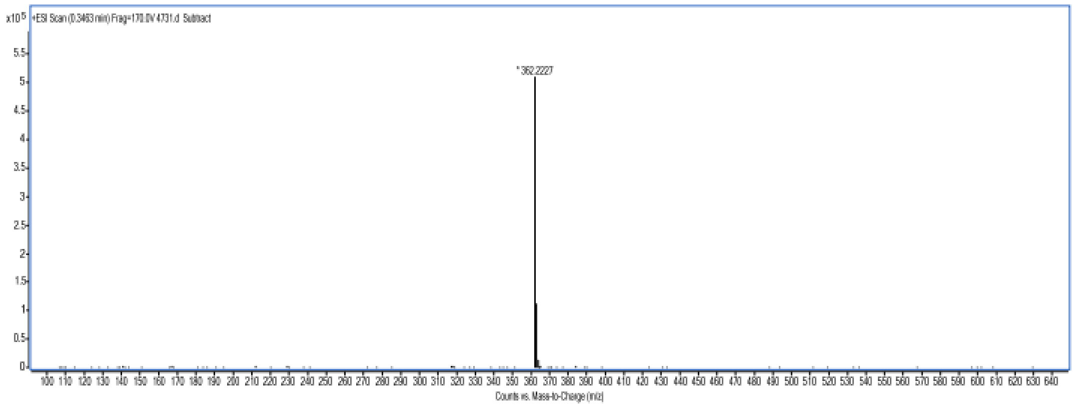

Figure S34 HR-ESI-MS of compound **6i**

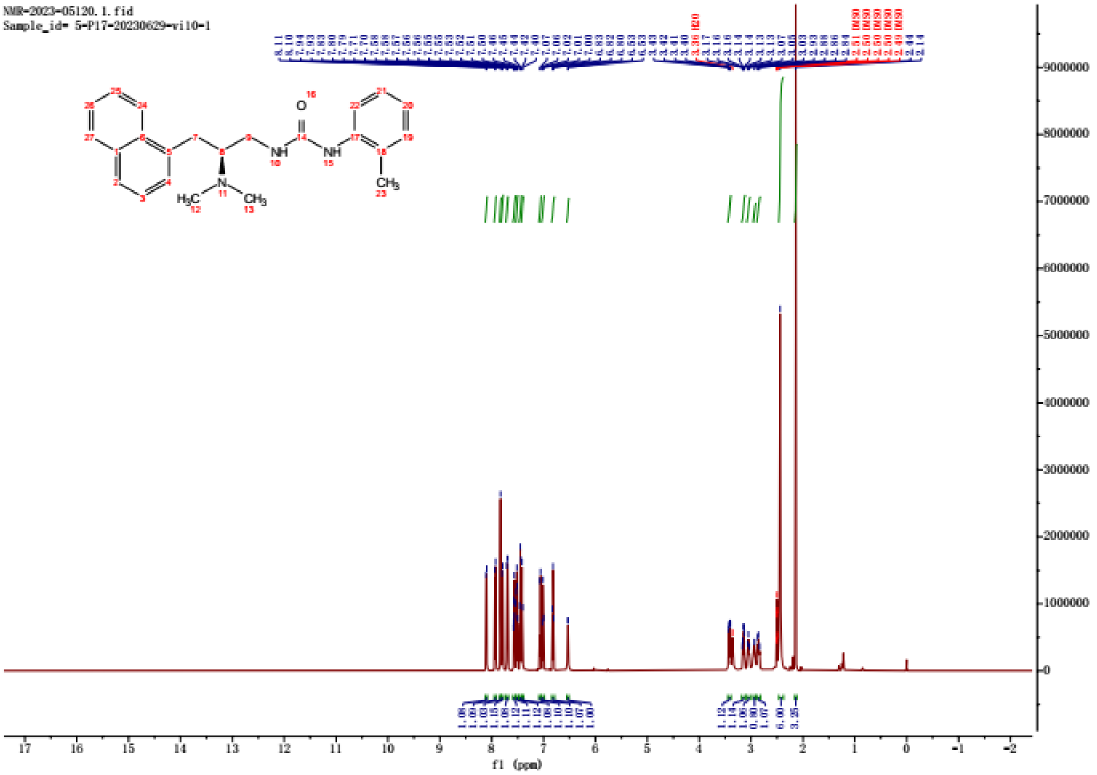

Figure S35 ^{1}H NMR spectra of compound **6i** in DMSO-d_6

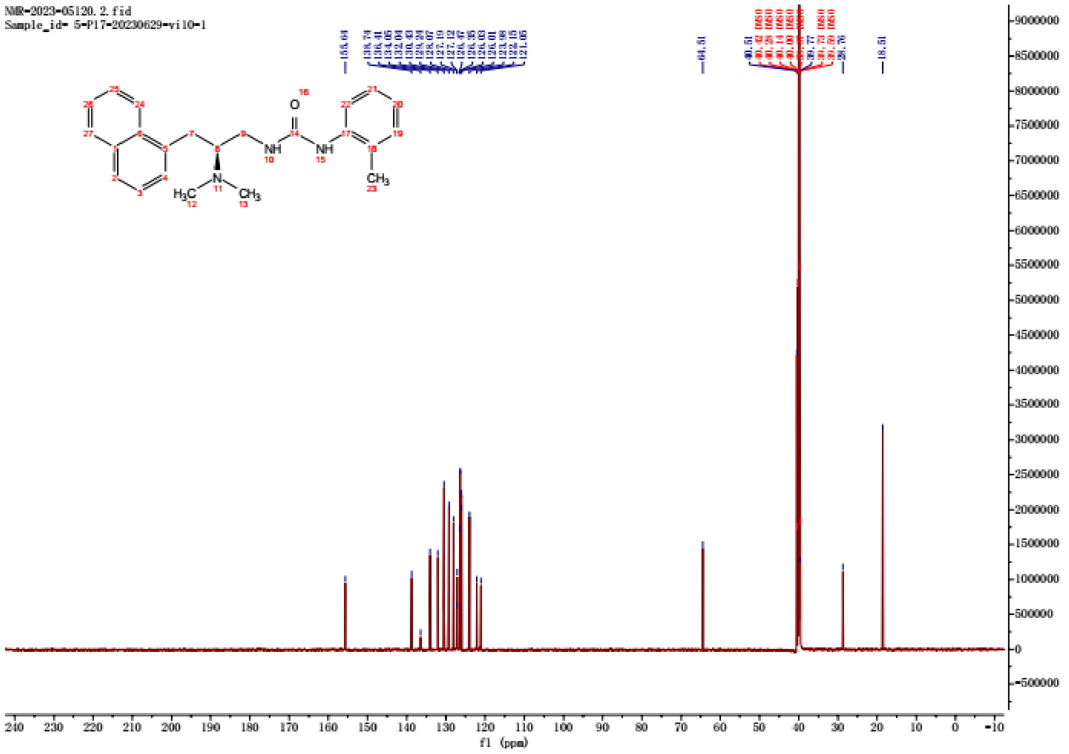

Figure S36 ^{13}C NMR spectra of compound **6i** in DMSO-d_6

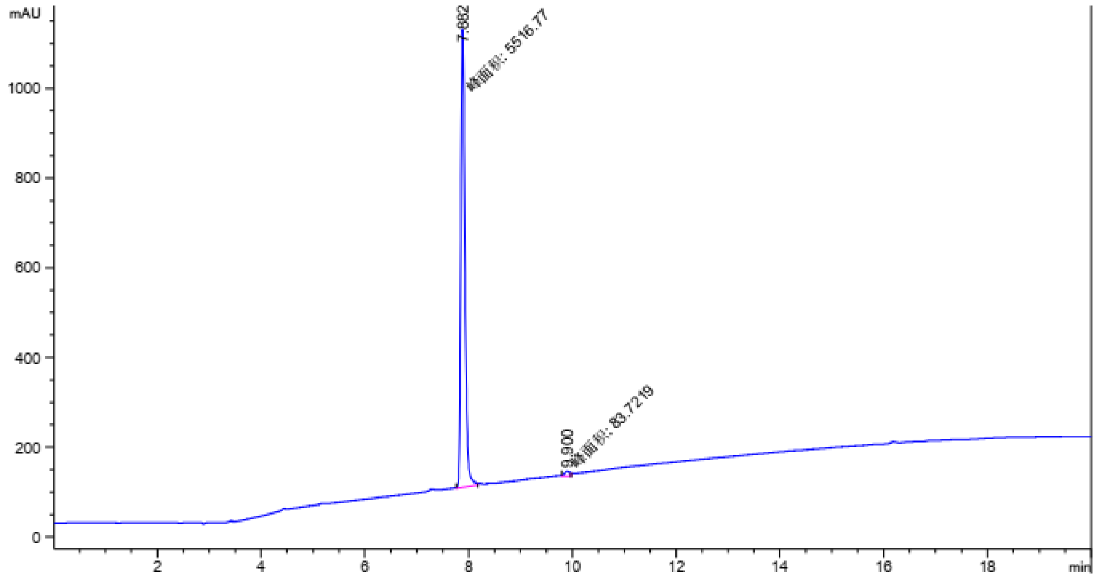

Figure S37 HPLC of compound **6j**

Qualitative Analysis Report

**Data Filename**	4732.d	**Sample Name**	5-P18-20230703-vi11-1
**Instrument Name**	TOF G6230A	**Acquired Time**	2023-07-20
**Acq Method**	YCL.M	**Acquired SW**	6200 series TOF/6500 series
**IRM Calibration Status**	Success		

User Chormatograms

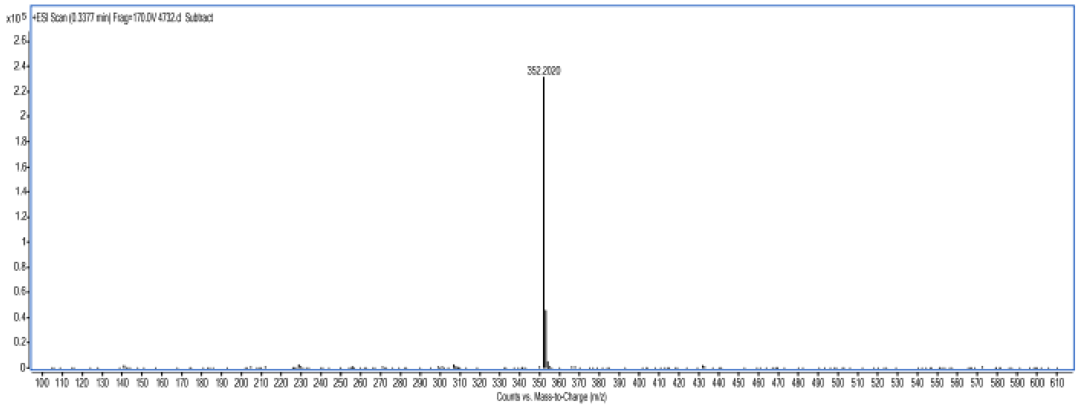

Figure S38 HR-ESI-MS of compound **6j**

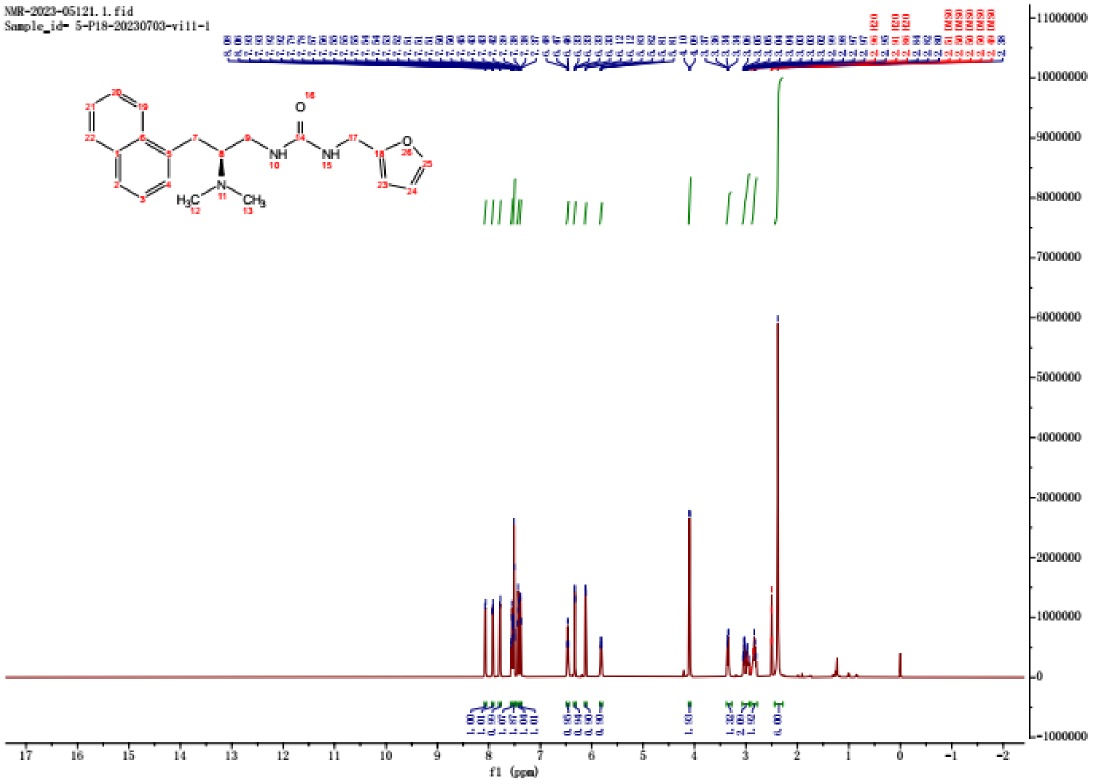

Figure S39 ^{1}H NMR spectra of compound **6j** in DMSO-d_6

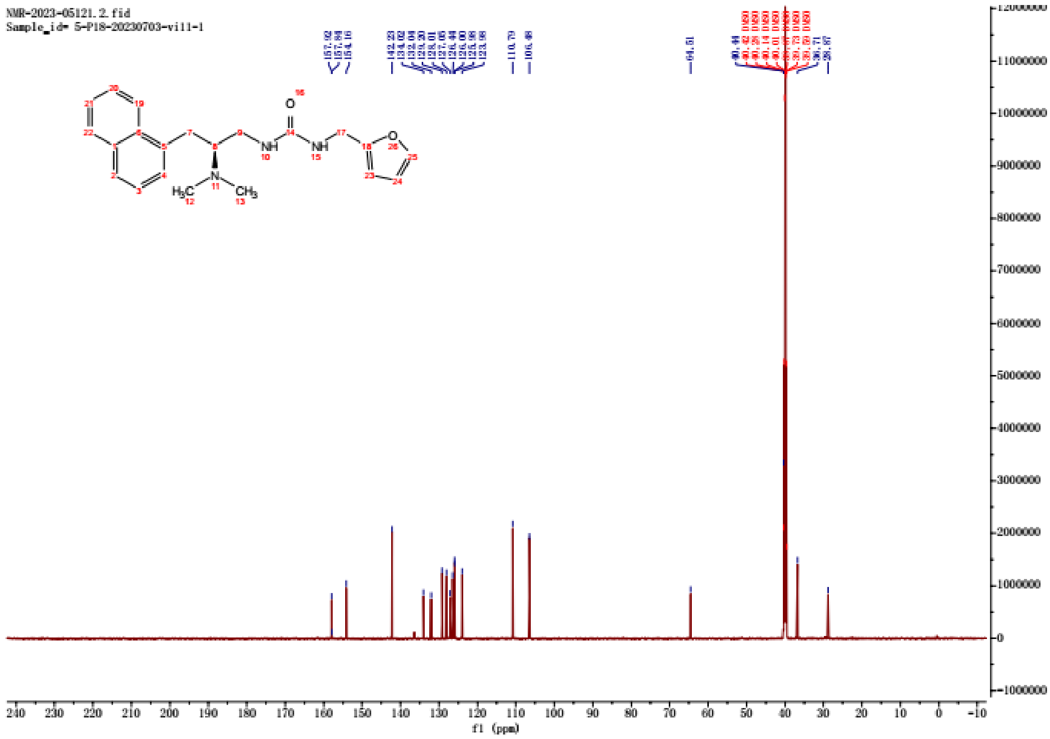

Figure S40 ^{13}C NMR spectra of compound **6j** in DMSO-d_6

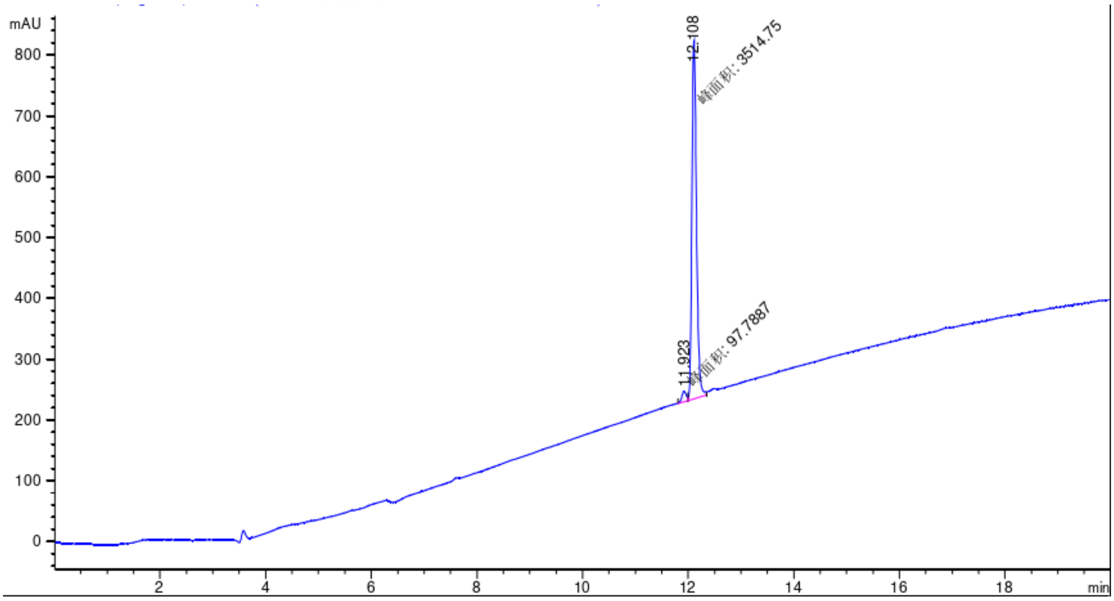

Figure S41 HPLC of compound **6k**

Qualitative Analysis Report

**Data Filename**	4733.d	**Sample Name**	5-P19-20230704-vi12-1
**Instrument Name**	TOF G6230A	**Acquired Time**	2023-07-20
**Acq Method**	YCL.M	**Acquired SW**	6200 series TOF/6500 series
**IRM Calibration Status**	**Success**		
**User Chormatograms**			

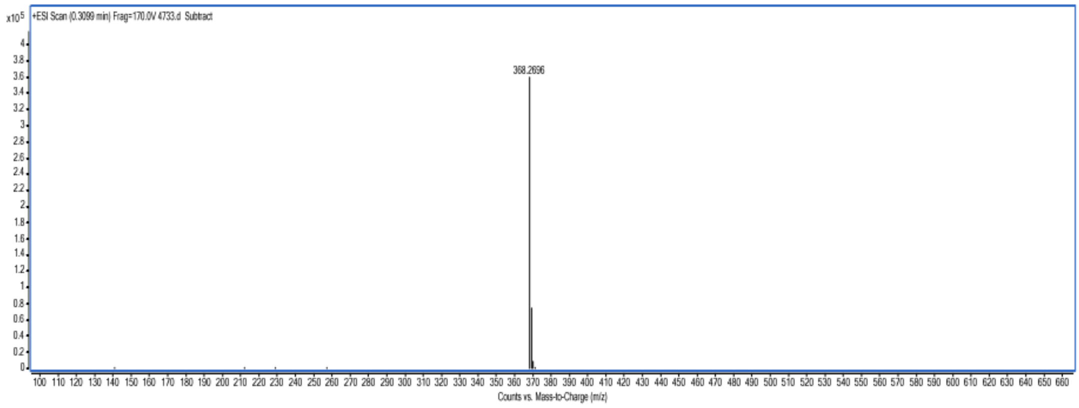

Figure S42 HR-ESI-MS of compound **6k**

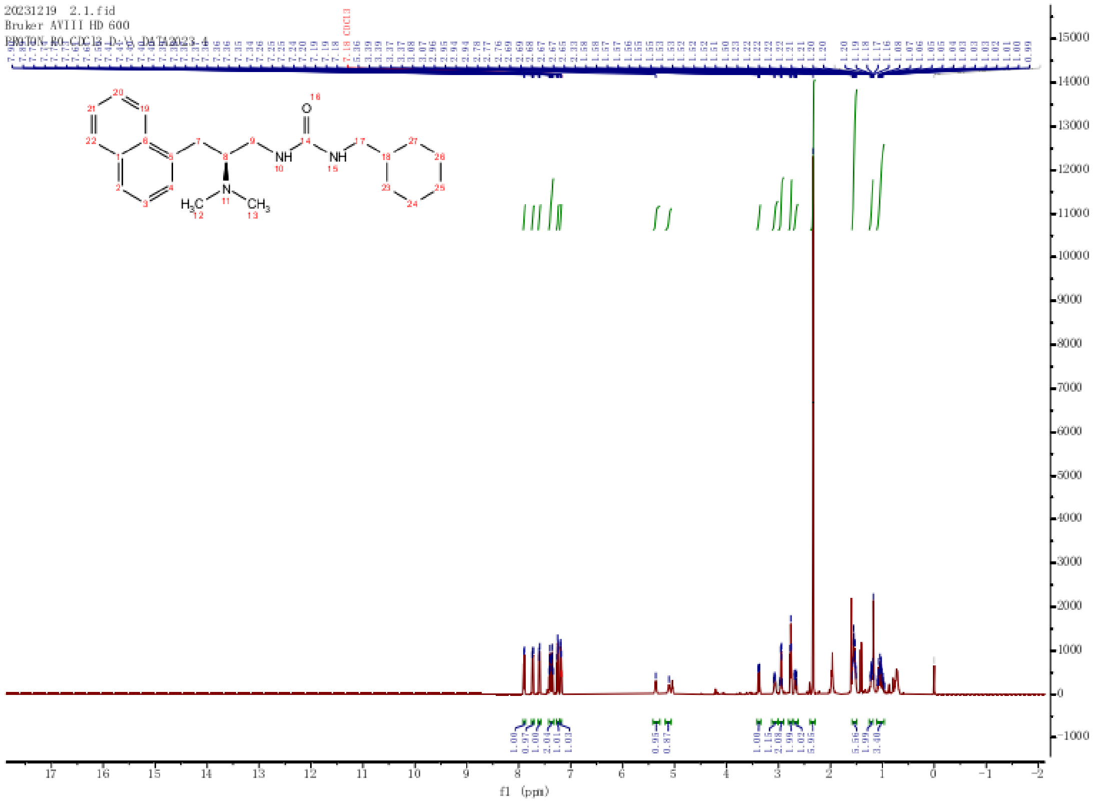

Figure S43 ^{1}H NMR spectra of compound **6k** in $CDCl_3$

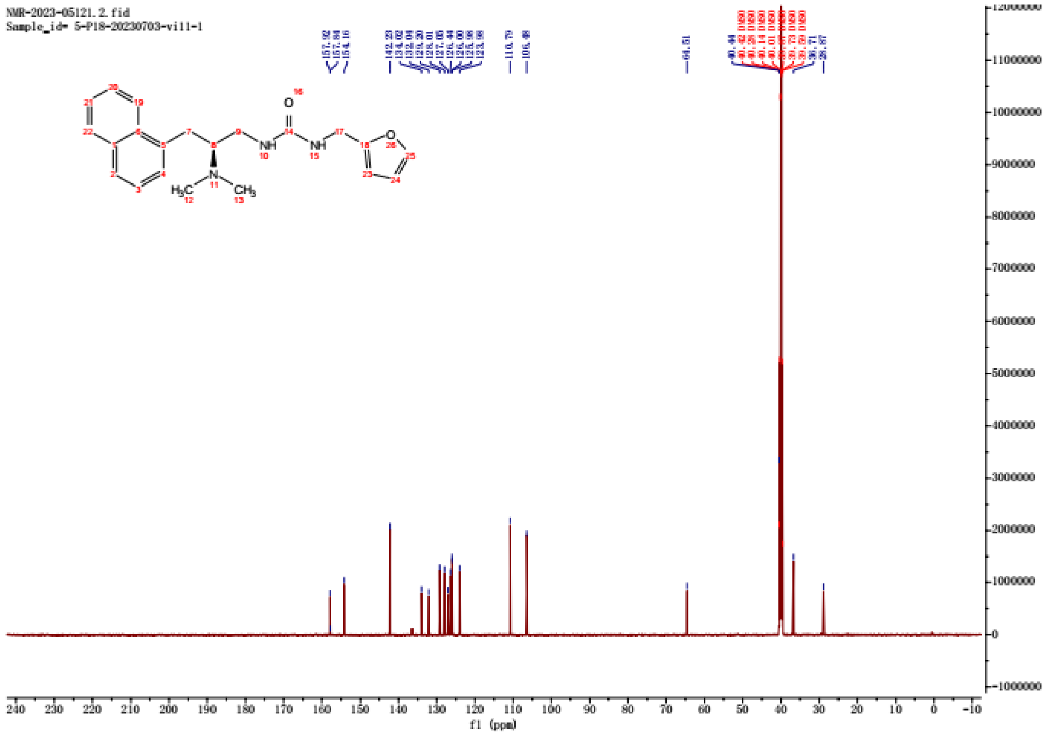

Figure S44 ^{13}C NMR spectra of compound **6k** in $CDCl_3$

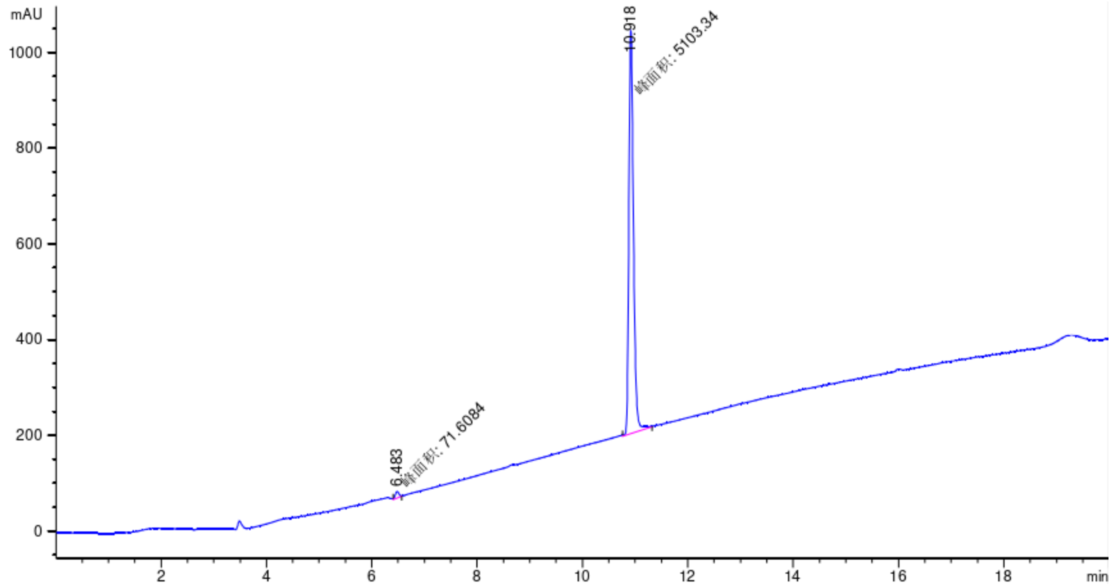

Figure S45 HPLC of compound **6l**

Qualitative Analysis Report

**Data Filename**	5208.d	**Sample Name**	5-P23-20230719-vi13-1
**Instrument Name**	TOF G6230A	**Acquired Time**	2023-08-15
**Acq Method**	YCL.M	**Acquired SW**	6200 series TOF/6500 series
**IRM Calibration Status**	**Success**		
**User Chormatograms**			

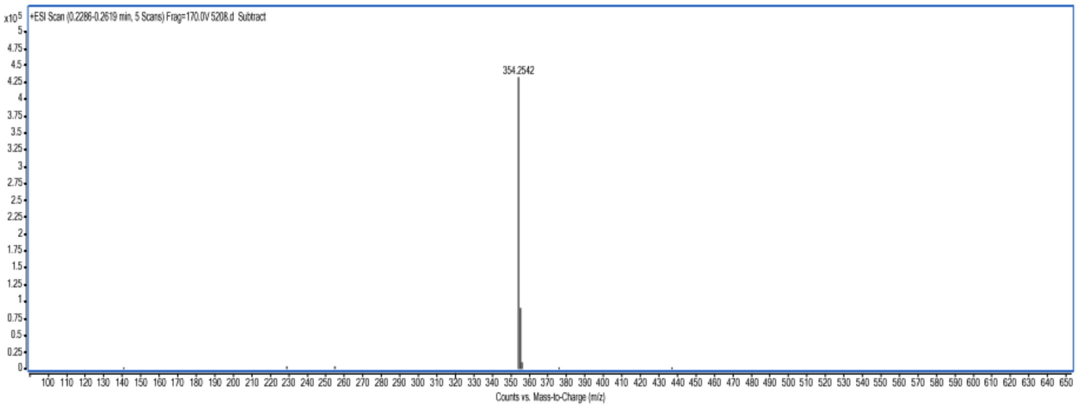

Figure S46 HR-ESI-MS of compound **6l**

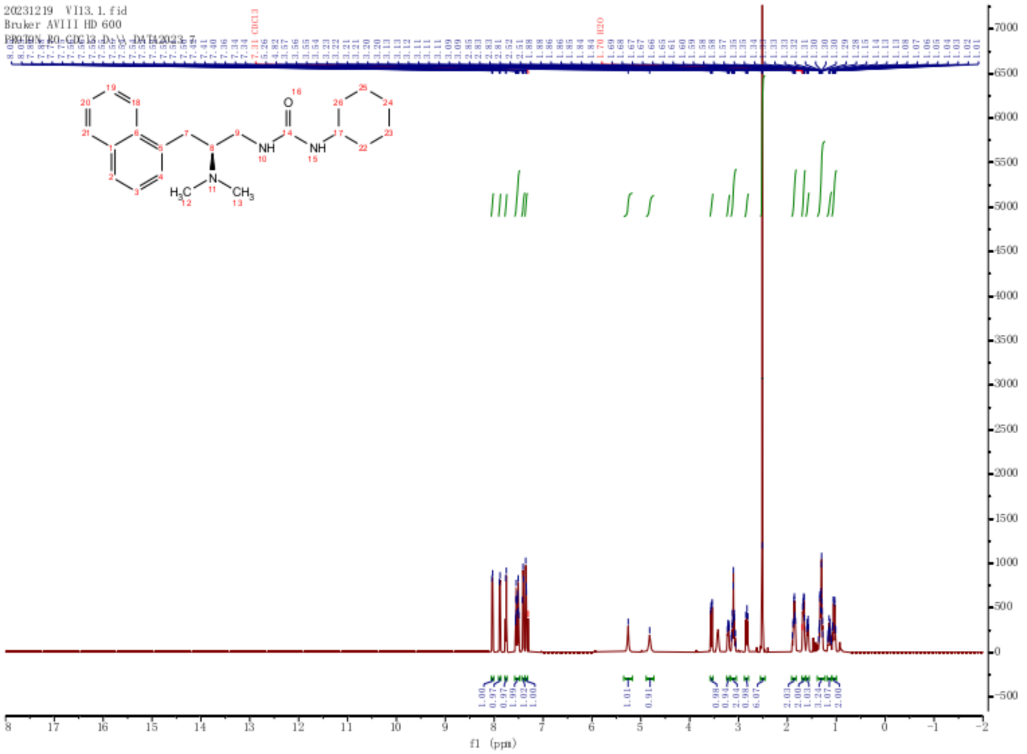

Figure S47 ^{1}H NMR spectra of compound **6l** in $CDCl_3$

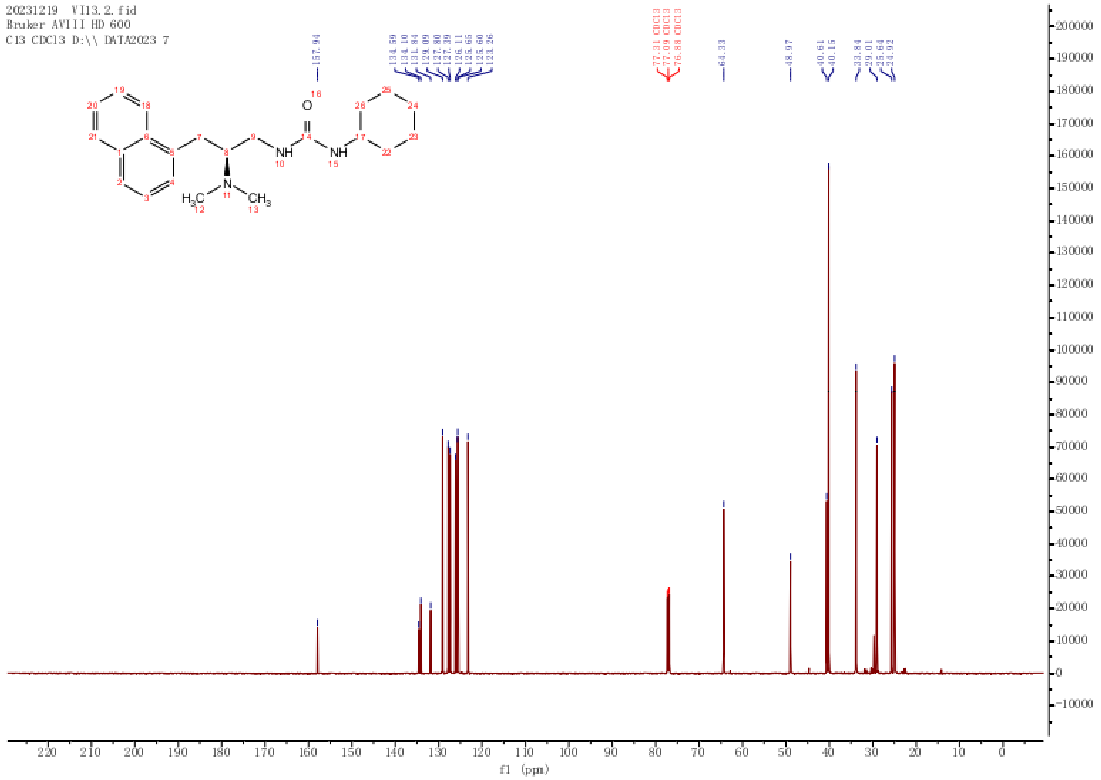

Figure S48 ^{13}C NMR spectra of compound **6l** in $CDCl_3$
